# Disambiguating brain functional connectivity

**DOI:** 10.1016/j.neuroimage.2018.01.053

**Published:** 2018-06

**Authors:** Eugene P. Duff, Tamar Makin, Michiel Cottaar, Stephen M. Smith, Mark W. Woolrich

**Affiliations:** a FMRIB Centre, Wellcome Centre for Integrative Neuroimaging, Nuffield Department of Clinical Neurosciences, University of Oxford, Oxford, OX3 9DU, United Kingdom; b Oxford Centre of Human Brain Activity, Wellcome Centre for Integrative Neuroimaging, Department of Psychiatry, University of Oxford, Oxford, OX3 7JX, United Kingdom; c Institute of Cognitive Neuroscience, University College London, WC1N 3AZ, United Kingdom; d Department of Paediatrics, University of Oxford, Oxford, OX3 7JX, United Kingdom

**Keywords:** Correlation, SNR, Functional connectivity, Effective connectivity, FMRI

## Abstract

Functional connectivity (FC) analyses of correlations of neural activity are used extensively in neuroimaging and electrophysiology to gain insights into neural interactions. However, analyses assessing changes in correlation fail to distinguish effects produced by sources as different as changes in neural signal amplitudes or noise levels. This ambiguity substantially diminishes the value of FC for inferring system properties and clinical states. Network modelling approaches may avoid ambiguities, but require specific assumptions. We present an enhancement to FC analysis with improved specificity of inferences, minimal assumptions and no reduction in flexibility. The Additive Signal Change (ASC) approach characterizes FC changes into certain prevalent classes of signal change that involve the input of additional signal to existing activity. With FMRI data, the approach reveals a rich diversity of signal changes underlying measured changes in FC, suggesting that it could clarify our current understanding of FC changes in many contexts. The ASC method can also be used to disambiguate other measures of dependency, such as regression and coherence, providing a flexible tool for the analysis of neural data.

## Highlights

• A new approach for interpreting functional connectivity (FC) analyses is presented.
• The approach identifies scenarios where changes may be attributed to changes in signal amplitude.
• The method helps to disambiguate FC measures.
• The general approach is applicable to analyses assessing changes in correlation and regression.

Correlation and regression are widely used to characterize the extent to which sets of signals are related, and how these relations might change over time or across experimental conditions. For example, functional connectivity (FC) analyses use correlation and related measures to identify networks of brain regions showing shared activity, to characterize differences within and between networks across different states (Friston, 1994, Friston, 2011, Cole et al., 2016, Smith et al., 2011, van den Heuvel and Hulshoff Pol, 2010, Shirer et al., 2012). FC methods include seed-region correlation (Biswal et al., 1995) psycho-physiological interaction (PPI) analysis (Friston et al., 1997, O'Reilly et al., 2012), data decomposition methods such as ICA (McKeown and Sejnowski, 1998, Beckmann and Smith, 2004, Cole et al., 2010), and network-matrix evaluations (Smith et al., 2013). These approaches can provide rich summaries of the large-scale patterns of synchronised brain activity, identifying distinct functional systems and their inter-relations. Differences in these patterns across states may indicate differences in inter-regional neural connectivity, and can be used for the decoding of brain and clinical states (Richiardi et al., 2011, Duff et al., 2013, Demertzi et al., 2015, Smith et al., 2015). Differences across subjects may be heritable (Colclough et al., 2017). However, correlation is sensitive to various changes in signal dynamics, making it an ambiguous marker of neural interactions (Friston, 1994, Friston, 2011, Power et al., 2012, Cole et al., 2016, Cohen and Kohn, 2011, Lee Rodgers and Nicewander, 1988). For example, changes in noise levels will alter correlation, as will changes in the amplitude of shared neural activity, or changes in other properties of the constituent signals (Friston, 1994, Friston, 2011, Cole et al., 2016). This ambiguity reduces the usefulness of correlation and related approaches for characterizing network structure and its alteration across states. This ambiguity furthermore substantially reduces the ability of FC to inform the specification and interpretation of more complex, multivariate modelling approaches that aim to distinguish directed and mediated relationships between nodes (Friston, 1994, Buchel, 1997, Marrelec et al., 2006, Smith et al., 2011, Costa et al., 2015). Similar challenges occur in the frequency domain, where analogues of correlation such as coherence can be influenced by a variety of very different changes in signal and noise properties (Cole et al., 2016).

Various approaches attempt to avoid the ambiguities of correlation and regression analyses. Effective connectivity approaches aim to distinguish direct from indirect connections, potentially removing the effects of noise and amplitude changes. These approaches include partial correlation (Smith et al., 2011, Marrelec et al., 2006, Duff et al., 2013), structural equation modelling (SEM) (Buchel, 1997), dynamic causal modelling (DCM) (Stephan et al., 2007, Daunizeau et al., 2012), Granger causality(Granger, 1969) and the distribution-based causal inference approach, LiNGAM (Shimizu et al., 2006). A variety of information may inform the estimation of these models, including the stationary or dynamic covariance structure, higher order moments, and timing discrepancies between regions. If appropriately defined, these approaches may avoid sensitivity to signal-to-noise ratio changes through their ability to model multiple sources of variance in the network. However, it is vital that the model assumptions are accurate, and the various signal and noise sources are appropriately modelled. Signal sources (e.g. measurements from distinct spatial regions) must be appropriately selected - mis-specified, missing, or redundant nodes can have a significant effect on network identification and estimation (Woolrich and Stephan, 2013). If complex network models do not appropriately account for major aspects of dynamics, missed effects can influence available network parameters in complex ways. Therefore, simpler descriptive approaches that identify the range of dynamics that need to be modelled are required to guide effective connectivity approaches.

Covariance is a key property for estimating several standard effective connectivity models, such as structural equation modelling and DCM. Direct assessment of covariance changes is a simple way to learn about the signals driving functional connectivity changes in variance and covariance. For example, increased temporal BOLD signal variance across the brain and skull may indicate that increased noise levels are affecting correlations. Evidence that the amplitude of fluctuations of functional signals can reflect important aspects of neural processes has been identified in neuroimaging and other neuroscience datasets (Duff et al., 2008, Friston, 2011, Zou et al., 2008, Cohen and Kohn, 2011, Faisal et al., 2008, Cole et al., 2016, Zöller et al., 2017). BOLD signal variance levels have been found to reliably correlate with behavioural parameters and clinical states (Duff et al., 2008, Zou et al., 2008, Cohen and Kohn, 2011, Fukunaga et al., 2008, Garrett et al., 2010) but may also reflect a host of non-neural sources, including physiological oscillations and imaging noise and artefacts (Cole et al., 2010, Van Dijk et al., 2012). Changes in signal volatility has been linked to dynamic changes in ongoing functional connectivity during rest (Preti et al., 2016, Lindquist et al., 2014, Eavani et al., 2013).

Despite the relationship of variance to functional connectivity, variance is not routinely characterized in conjunction with correlation. One reason for this is a lack of quantitative approaches that integrate correlation and variance. In recent work, Cole et al. have focused on how covariance can inform the assessment of changes in functional connectivity, describing a conjunction-based approach combining correlation and covariance changes to provide insight into possible changes in amplitude of shared or unshared signals underlying FC changes (Cole et al., 2016). With simulations and empirical data, they show how changes in FC can be driven by the introduction of signal shared across nodes. The proposed conjunction approach identifies when correlation changes are accompanied by covariance changes, and therefore could be explained by changes in the amplitude of shared signal. However, this approach does not determine whether the variance changes are adequate to explain the change in correlation.

Here we present a covariance-based approach for the analysis of changes in functional connectivity, which provides enhanced inferences relating to the nature of the changes in signal across different states with minimal additional assumptions. The approach identifies Additive Signal Changes (ASC), a class of changes involving simple additions of new signal to the existing signal. This class encompasses a variety of natural phenomena that will occur in many systems, including simple changes in noise levels, and changes in the amplitude of a common signal driving correlation between two nodes. Changes in signals that do not conform to additive changes include those involving more complex combinations of increases and decreases in the strength of existing signal components, or the wholesale synchronisation of activity across two nodes. A key feature of the ASC class is that correlation changes are always accompanied by changes in variance. This makes it possible to perform null-hypothesis tests regarding whether changes in variance are adequate for pure ASC changes to explain observed FC changes. Thus, the ASC approach extends the methods described by Cole et al. (2016), providing direct null-hypotheses inferences regarding putative properties of underlying signal changes. This enables analysts to determine whether certain major classes of change may explain observed FC changes. Like the methods described by Cole et al., the approach requires only the correlation and (co)variance of signals.

The ASC approach is related to causal network-inference approaches like structural equation modelling and dynamic causal modelling, but focuses on providing a flexible bivariate analysis that provides insight into general properties of the signal dynamics, permitting rapid exploratory analyses with limited assumptions. Applied to FMRI experimental data, we find that this approach provides valuable additional insight into patterns of functional connectivity modulations. The results suggest caution for interpreting FC changes purely as changes in coupling, as we find a substantial proportion of correlation changes can be explained by changes in the amplitude of existing signals. As the approach employs a model that is closely related to effective connectivity methods, it also provides a valuable link between functional and effective connectivity approaches.

In the following, we describe the ASC approach and inference procedures, with a focus on network matrix FC analyses. We then test the approach on simulated and empirical data. Our empirical dataset assesses differences between rest and different ongoing “active state” functional states in individuals. These states were designed to produce a mix of robust, localised changes in FC to provide a good test bed for assessing the value of ASC analysis for disambiguating FC changes. Finally, we discuss additional potential applications of the ASC approach, and how these analyses may contribute to more comprehensive analyses of functional and effective connectivity in neuroimaging.

## Materials and methods

### Model

We are interested in making inferences regarding the source of observed changes in the correlation of two signals across different states (e.g. cognitive or disease states). Consider nodes *X* and *Y* (e.g. brain regions), producing signal whose correlation we measure across two conditions, *A* and *B*. $X_{A}$ and $Y_{A}$ represent the signals in nodes *X* and *Y* in condition *A*. We assume these signals are produced by an ergodic stochastic process providing a covariance matrix ${\text{Σ}}_{X_{A},Y_{A}}$:

$${\text{Σ}}_{X_{A},Y_{A}}=\left[\begin{array}{cc} \sigma_{X_{A}}^{2} & \sigma_{X_{A},Y_{A}}\\ \sigma_{X_{A},Y_{A}} & \sigma_{Y_{A}}^{2} \end{array}\right]$$

Here, $\sigma_{X_{A}}^{2}$ is the variance of node *X* in state *A*, and $\sigma_{X_{A},Y_{A}}$ is the covariance between nodes. Estimates from data of changes in covariance across states ($Q_{A}$, $Q_{B}$) are used for inferences about the nature of the change in underlying processes across the conditions.

### Defining additive changes in signal

Our analysis approach focuses on determining whether differences in the correlation between nodes *X* and *Y* across states *A* and *B* can be explained by Additive Signal Changes (ASC), a general class of changes in the nature of the stochastic process. In node *X*, a change from state *A* to state *B* is an ASC if the change can be described by the addition of new signal:

$$X_{B}=X_{A}+X_{N},\;\text{where},\;\rho_{X_{A},X_{N}}\geq 0$$

The intuition here is that an additive change does alter the initial signal, $X_{A}$. Additive changes are a natural class of signal change, corresponding to scenarios where a new component of signal is added to the existing signal. Equally, it encompasses scenarios where one or more components increase in strength in one state, while other signal components remain unchanged. The class excludes changes which may involve the initial signal being modified, replaced, or changing in sign. A key property of ASCs is that they always produce a change in variance: $\sigma_{X_{B}}^{2}>\sigma_{X_{A}}^{2}$.

As a characterization of changes between two states, ASC is directional. If a change from state A to B is additive, it is not additive from B to A. The change between two states is additive in one direction if (see Supplementary Materials and Methods):

$$\sigma_{X_{N}}^{2}\leq \left|\sigma_{X_{B}}^{2}-\sigma_{X_{A}}^{2}\right|$$

A change in functional connectivity between two nodes implies a change in the nature of the signal in at least one of these nodes. We are interested in inferring whether the changes in FC of two nodes *X* and *Y* across states can be explained by additive changes in signal in either of the two nodes, thus identifying or excluding a variety of signal changes as possible causes of the observed changes in functional connectivity. Changes in FC due to ASC changes can be expected to occur in many contexts (Figs. 1.1-1.3). For example, an increase in uncorrelated noise levels results in additive increases in uncorrelated signal variance in both nodes, reducing correlation. Conversely, an increase in amplitude of some process shared across nodes can increase correlation. An ability to identify when specific additive effects can explain observations would be valuable for interpreting functional connectivity.

**Fig. 1:**
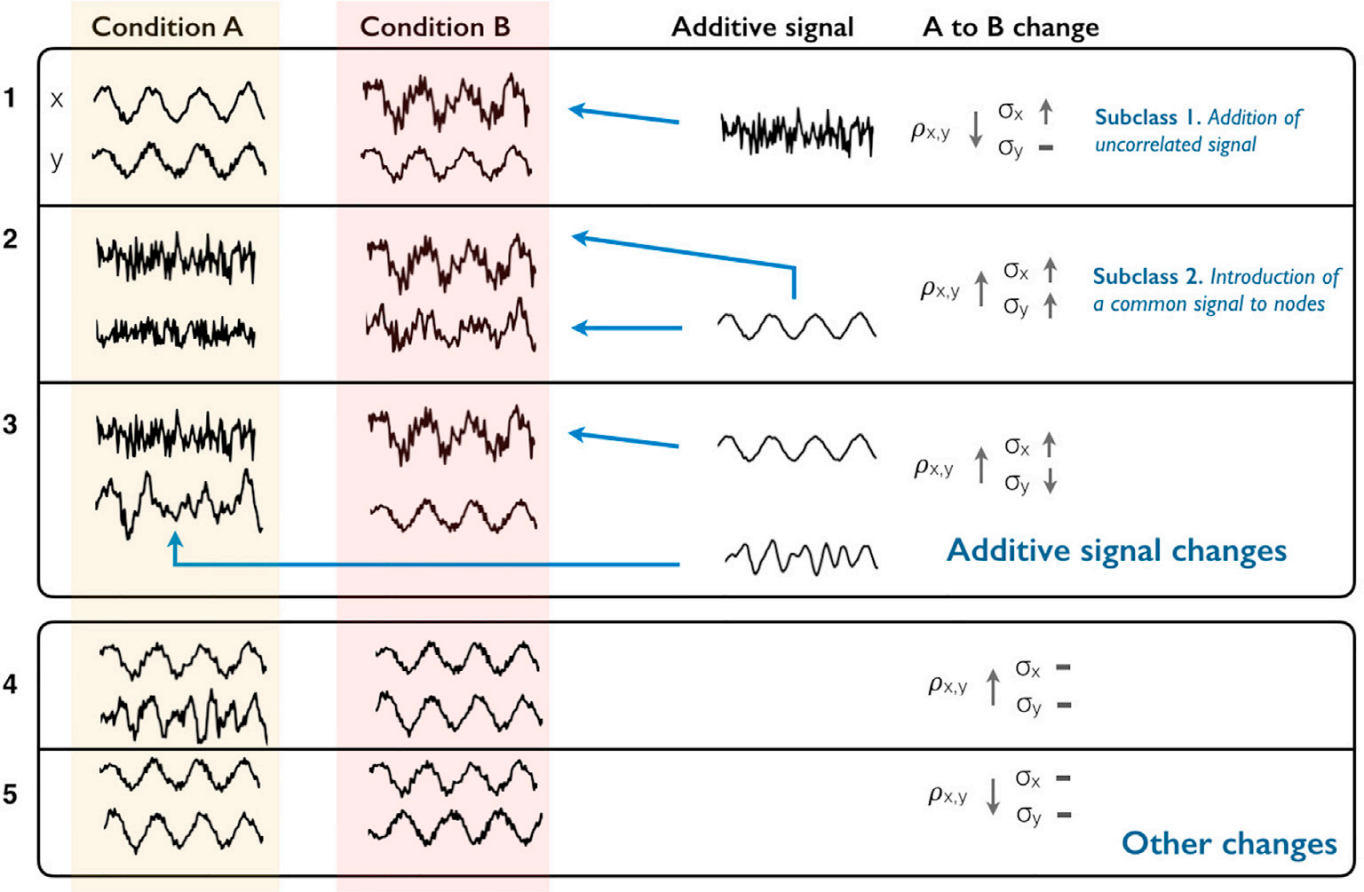
Representation of effects of additive signal changes (ASC) on correlation. The upper box demonstrates three examples of additive signal changes to correlation. The blue arrows represent the addition of signal into a node in a certain state. The first example corresponds to the subclass of additions in uncorrelated signal. Here, signal uncorrelated with region *Y* is added to *X* in state *B*, reducing correlation and increasing variance. In the second, a common signal is added to both regions in state *B*, increasing correlation and variance. In the third example, region *X* receives an addition of signal already present in region *Y*. At the same time, some signal not shared by region *X* (e.g. some input from a third region) is removed from region Y in state *B*. The overall effect is an increase in correlation. The second box shows some scenarios that do not fall within the class of additive changes. The first example shows a synchronisation of signals whose temporal properties, including variance, otherwise do not substantially change. The final example shows two signals where their correlation flips from positive to negative. This could be explained by the addition of a great deal of negatively correlated signal, but falls outside our definition of additive signal.

For a change in FC to fall into our Additive Signal Change class, we require only that the change could be explained by some type of additive change: we place no restrictions on the relationship of the additive signals across the two nodes, nor require that the additive signal occurs in the same state. This implies the following inequalities:

$$X_{B}=X_{A}+X_{N},\text{where},\{\begin{array}{cc} \rho_{X_{A},X_{N}}\geq 0 & \text{if }\sigma_{X_{B}}^{2}\geq \sigma_{X_{A}}^{2}\\ \rho_{X_{B},-X_{N}}\geq 0 & \text{otherwise}. \end{array}$$

$$Y_{B}=Y_{A}+Y_{N},\text{where},\{\begin{array}{cc} \rho_{Y_{A},Y_{N}}\geq 0 & \text{if }\sigma_{Y_{B}}^{2}\geq \sigma_{Y_{A}}^{2}\\ \rho_{Y_{B},-Y_{N}}\geq 0 & \text{otherwise}. \end{array}$$

Which is equivalent to:

$$\sigma_{X_{N}}^{2}\leq \left|\sigma_{B_{X}}^{2}-\sigma_{X_{A}}^{2}\right|$$

$$\sigma_{Y_{N}}^{2}\leq \left|\sigma_{Y_{B}}^{2}-\sigma_{Y_{A}}^{2}\right|$$

While different additive changes will have different effects on correlation, additive changes producing substantial change in correlation will produce relatively large additions of signal. As ASCs always produce variance changes, it is possible to determine whether observed changes in variance are adequate for additive changes to explain a given change in correlation. From the initial correlation, and changes in variance, we can estimate the range of values of the final correlation, $\rho_{X_{B},Y_{B}}$, that could be explained by additive signals. We can express $\rho_{X_{B},Y_{B}}$ as:

(1) $$\begin{array}{c} \rho_{X_{B},Y_{B}}=\rho_{X_{A}+X_{N},Y_{A}+Y_{N}}\\ =\frac{\sigma_{X_{A}+X_{N},Y_{A}+Y_{N}}}{\sigma_{X_{A}+X_{N}}\sigma_{Y_{A}+Y_{N}}}\\ =\frac{\sigma_{X_{A},Y_{A}}+\sigma_{X_{A},Y_{N}}+\sigma_{X_{N},Y_{A}}+\sigma_{X_{N},Y_{N}}}{\sigma_{X_{B}}\sigma_{Y_{B}}}\\ =\frac{\sigma_{X_{A},Y_{A}}+\rho_{X_{A},Y_{N}}\sigma_{X_{A}}\sigma_{Y_{N}}+\rho_{Y_{A},X_{N}}\sigma_{Y_{A}}\sigma_{X_{N}}+\rho_{Y_{N},X_{N}}\sigma_{X_{N}}\sigma_{Y_{N}}}{\sigma_{X_{B}}\sigma_{Y_{B}}} \end{array}$$

We are interested in finding the minimum and maximum values of $\rho_{X_{B},Y_{B}}$ given 5 unknowns: $\sigma_{X_{A},Y_{N}}$, $\sigma_{Y_{A},X_{N}}$, $\rho_{X_{A},Y_{N}}$, $\rho_{Y_{A},X_{N}}$, and $\rho_{X_{N},Y_{N}}$, with constraints:

$$\begin{array}{c} \sigma_{X_{A}}^{2}+\sigma_{X_{N}}^{2}+2\rho_{X_{A},X_{N}}\sigma_{X_{A}}\sigma_{X_{N}}=\sigma_{X_{B}}^{2}\\ \sigma_{Y_{A}}^{2}+\sigma_{Y_{N}}^{2}+2\rho_{Y_{A},Y_{N}}\sigma_{Y_{A}}\sigma_{Y_{N}}=\sigma_{Y_{B}}^{2}\\ \sigma_{X_{N}}^{2}\leq |\sigma_{X_{B}}^{2}-\sigma_{X_{A}}^{2}|\\ \sigma_{Y_{N}}^{2}\leq |\sigma_{Y_{B}}^{2}-\sigma_{Y_{A}}^{2}| \end{array}$$

The problem can be reformulated in terms of orthogonal components, and can be solved by solving the Karush-Kuhn-Tucker (KKT) conditions (see Supplementary Materials and Methods). Once the range of changes in correlation that can be explained by ASCs is determined, we can use this to test whether the observed difference between covariances could be explained by this class of change. In practise, hypothesis testing must accommodate observation error. Our approach for this is described below.

We can define subclasses of ASC and derive tests for whether these specific effects can explain observed changes in FC. These subclasses place additional constraints on the nature of the additive signals:

1. *Addition of uncorrelated signal.* Here additive processes are uncorrelated with processes in the other node. That is, $X_{N}$ is uncorrelated with both $Y_{B}$ and $Y_{N}$, and $Y_{N}$ is uncorrelated with $X_{A}$ and $X_{N}$ (see Fig. 1.1). This subclass covers scenarios such as changes in levels of uncorrelated measurement noise, and changes in the amplitude of signal components that are independent of activity in the second node. If the additive signal is uncorrelated in this way, then Eqn. (1) becomes:

(2) $$\rho_{X_{B},Y_{B}}=\frac{\sigma_{X_{A}}\sigma_{Y_{A}}}{\sigma_{X_{B}}\sigma_{Y_{B}}}\rho_{X_{A},Y_{A}}$$

For this scenario, given changes in variance will predict a specific change in correlation (excluding observational uncertainties), with increases in correlations predominately accompanied by decreases in variance (2).

2. *Introduction of a common signal to both nodes.* This class corresponds to the scenario where there is an addition of the same latent signal component to both nodes, i.e., we set $\rho_{X_{N},Y_{N}}=1$ (Fig. 1.2). Here, in Eqn. (1), we can set $X_{N}=s_{X_{N}}N_{c},Y_{N}=s_{Y_{N}}N_{c}$ , where $N_{c}$ is a latent stochastic process set to have variance of 1, and $s_{X_{N}}$ and $s_{Y_{N}}$ are scaling factors, such that $\sigma_{X_{N}}=s_{X_{N}}$ and $\sigma_{Y_{N}}=s_{Y_{N}}$. The effect of the common signal will depend on the new signal's relationship to existing signals ($\sigma_{X_{A},N_{c}},\sigma_{Y_{A},N_{c}}$):

$$\rho_{X_{B},Y_{B}}=\frac{\sigma_{X_{A},Y_{A}}+\sigma_{X_{A},N_{c}}+\sigma_{Y_{A},N_{c}}+s_{X_{N}}s_{Y_{N}}}{\sigma_{X_{B}}\sigma_{Y_{B}}}$$

As for the ASC class, additions of a common signal can produce a variety of changes in correlation, and always produces a change in node variances. We can the calculate maximum and minimum correlation changes produced by common signals for an observed change in variance using KKT conditions (see Supplementary Materials and Methods). As the class is a subset of ASC, the range of potential correlation changes will be smaller than the range that can be explained by the general ASC class. While additions of uncorrelated signal reduce correlation, an addition of common signal will typically produce an increase in correlation.

*Negative correlations* The ASC analysis is applicable to both positively and negatively correlated signals. For negatively correlated signals, we define Common signals to have opposite signs in the two nodes, such that increases in common signal tend to increase the absolute correlation between nodes.

### Statistical inference

**Fig. 2:**
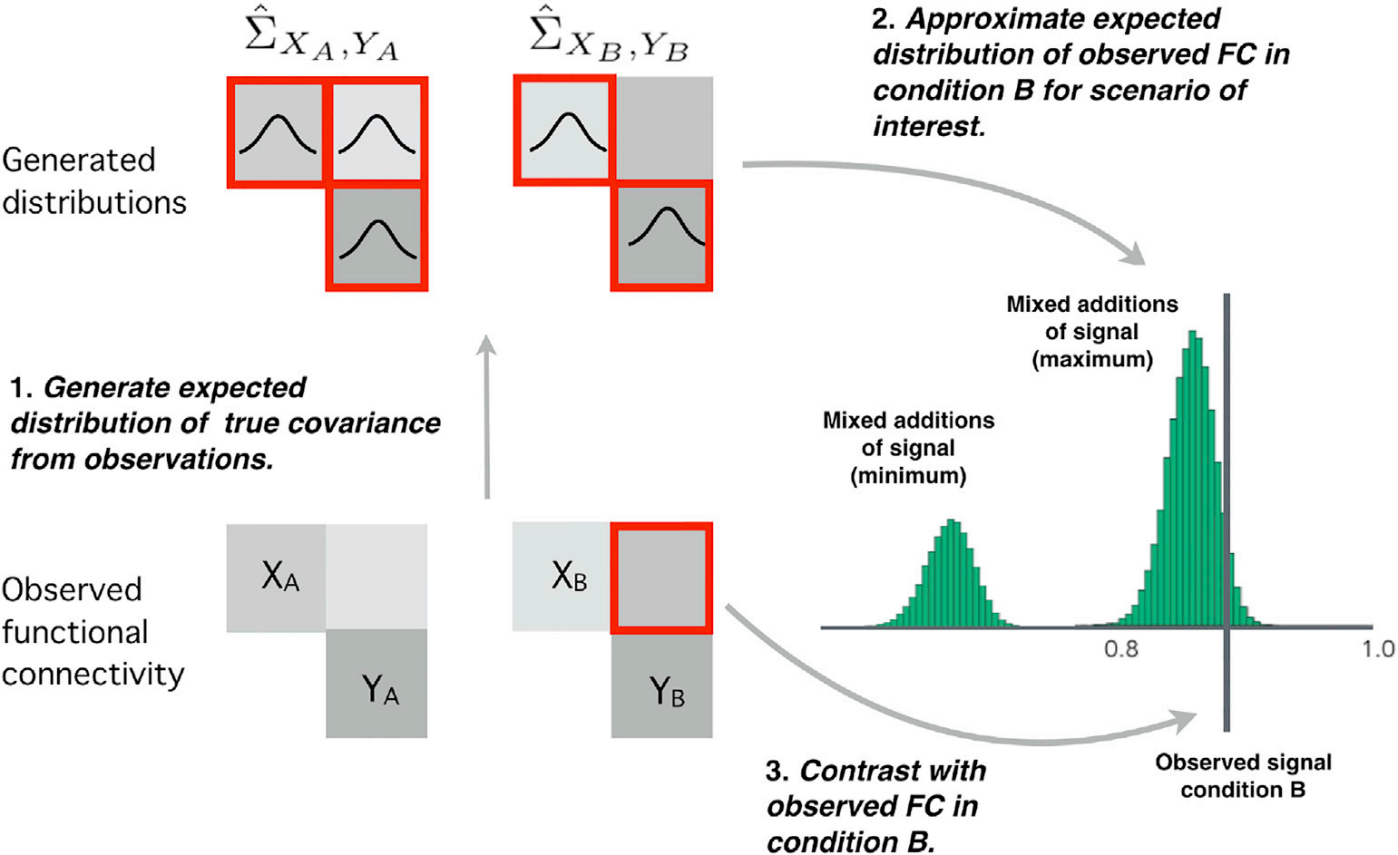
The Monte Carlo procedure used for inference on changes in functional connectivity. 1. From the observed covariances a distribution of potential underlying true covariances is generated, using a Wishart distribution and rejection sampling. 2. From these samples, the expected distribution of observed correlation for different additive signal change scenarios can be calculated (green histogram). These distributions can be compared to the observed correlation in state *B*, which can be used to test the hypothesis that the observed data is explained by a given scenario. Note that the common and general additive signal classes cover a range of different putative additive signals, which can have a range of effects on correlation. We identify those signals from these classes that will produce the minimum and maximum correlation, and use these signals to generate distributions for minimum and maximum possible observed correlations for these classes. 3. The observed FC is compared to these distributions to test null hypotheses that additive signal changes can explain observations.

FC analyses typically make inferences regarding whether observed correlations indicate significant differences across states. The ASC model permits additional inferences regarding the possible nature of the signal changes underlying these changes. We take a Monte Carlo (MC)-based null-hypothesis approach, assessing the likelihood that additive signal changes, and the subclasses defined above, would produce the observed changes in correlation and variance. This approach takes into account the imprecision of measured covariances by sampling from a distribution of underlying putative true covariances (Fig. 2).

We first sample from the distribution of possible underlying covariances of the observed signals (${\text{Σ}}_{X_{A},Y_{A}},{\text{Σ}}_{X_{B},Y_{B}}$) by rejection sampling using inverse Wishart distributions (see Supplementary Material, *Monte-Carlo Inference*). This assumes a Gaussian generative signal and a flat prior. Appropriate degrees of freedom (dof) for the autocorrelated time fMRI series is estimated by fitting AR signal models to the signal (Fig. 2).

To test whether observed changes in covariance, $Q_{A}$, $Q_{B}$, could be produced by additions of uncorrelated signal (ASC subclass 1), we first sample from the distribution of underlying true covariances of state *A*, the distribution of true variances in both states, given the observed signal. We then use Eqn. (2) and a Wishart distribution to generate samples of expected observed correlations in the second state. From these distributions we can identify an interval for the expected range of correlation given an addition of uncorrelated signal. If the observed correlation in the second state falls outside of this range, we infer that uncorrelated is unlikely to have produced this observation.

A similar approach is used to determine whether changes in covariance can be explained by an addition of a common signal component (ASC subclass 2), and by other additive signal changes. For these classes, the change in correlation depends on the exact nature of the introduced signals, which is unknown. However, we can determine those putative common and additive signals that produce the greatest possible changes in correlation (increases or decreases). To perform inference, those signals identified as producing the maximum and minimum possible changes in correlation are used in the sampling approach described above, to produce separate distributions for the expected maximal and minimal value of correlations that can be explained in these scenarios (Fig. 2). The minimum and maximum distributions are used to separately test for whether the observed correlation falls below the minimum, or above the maximum correlation, that can be explained by the common/additive classes. Note that this is a conservative procedure: most common or additive signals would produce smaller changes in correlation. For example, we reject the null hypothesis that an increase in correlation is produced by a common signal if the observed change in correlation is greater than the upper limit of the confidence interval for the correlation produced by the common signal that would produce the largest possible increase in correlation. As the class of additive signals encompasses the uncorrelated and common signal classes, the interval of correlations falling within the null hypothesis for additive signals this class will encompass the two subclasses.

In our demonstrative application of the above procedures, we assessed multiple connections within a network of brain regions. To present the results, we first identified connections showing a significant change in correlation (corrected the using False Discovery Rate, α=0.2). These are presented in four connectivity charts, showing changes in correlations that: 1. can be explained by uncorrelated signal, 2. can be explained by common signal, 3. can be explained by other additive signal changes, or 4. cannot be explained solely by additive changes of signal. Membership of these classes was determined using the MC approach above with 2000 iterations. Data was presented using code adapted from the MNE suite (www.martinos.org/mne) (Gramfort et al., 2014).

### Experimental methods

**Fig. 3:**
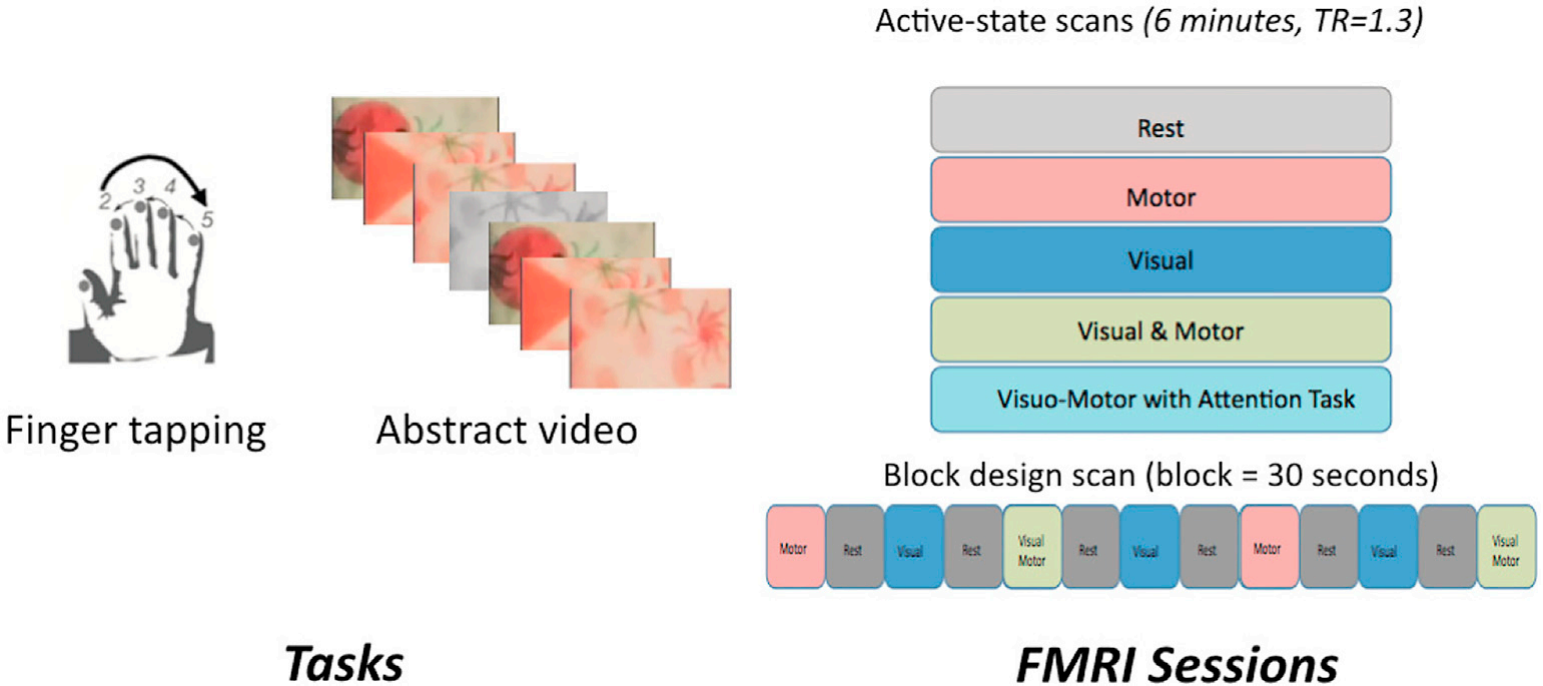
**Validation experiment.** A. Steady state tasks involved one or both of continuous fingertapping and viewing of a rapidly changing random images. The fingertapping had a consistent order, which was periodically reversed. B. Five 6-min steady state conditions were used. In the Visual & Motor condition subjects simply performed the motor task while viewing the video. In the attention task condition subjects were cued to change direction when specific visual cues were observed in the video.

We assessed the extent to which the ASC approach informs functional connectivity analyses by applying it to simulated data, and to an FMRI study of functional connectivity changes across resting and steady-state motor and visual task conditions (Costa et al., 2015). Sixteen healthy volunteers were scanned under five separate five-minute steady-state conditions, with no baseline epochs: rest (eyes open), visual only, motor only, simultaneous (but independent) visual and motor tasks, and a combined condition involving a visually-cued motor task (Fig. 3). The visual conditions consisted of videos of colorful abstract shapes in motion. The motor conditions involved continuous and monotonic sequential finger tapping against the thumb, using the right hand. The combined motor conditions combined the visual stimulus with finger tapping. In the visually-cued motor task subjects where instructed to change tapping direction when they saw an irregularly appearing cue, which were present in all visual conditions. These conditions were designed to induce robust changes in functional connectivity within well defined networks. An additional block-design task-activation localizer FMRI scan was performed under the same conditions to enable the identification of brain regions changing in average activation levels during these conditions. This scan used pseudo-randomised 30-s block intervals separated by 30-s baseline periods. One initial participant was discarded from analysis, due to an error in the block design acquisition. Data was acquired in a Siemens 3T scanner, using a 32-channel coil and a high-resolution ($2mm^{3}$) fast (TR = 1.3s) multiband (factor 6) whole-brain acquisition (Feinberg et al., 2010, Moeller et al., 2010). Scans were five minutes (230 time points).

We generated a set of 33 focal, task-relevant ROIs associated with activations and deactivations in the block design localizer scan (Supplementary methods, Table 1). To increase specificity, we targeted regions activated or deactivated in the task localizer scans. To identify task-related regions, we delineated regions identified as significantly activated in the localizer task-activation analyses (using an F-test across conditions, see Supplementary Material). To separate nodes, these regions were intersected with the Harvard-Oxford parcellation of the cortex (www.fmrib.ox.ac.uk/fsl). The ROIs were generated by intersecting the Harvard-Oxford atlas (www.fmrib.ox.ac.uk/fsl) with the thresholded activation map derived from a group-level F-test of the combined condition task in the localizer scan (Supplementary methods). The activations were highly symmetric except for those in the motor system, associated with the unilateral motor task. To enable us to explore the extent to which resting state symmetry of FC was preserved in the context of asymmetric motor activations we symmetrised these activations when generating the ROIs. We divided the ROIs into three broad classes, those associated with: 1. Visual activation 2. Motor activation, and 3. Deactivations. This mask is available at: http://neurovault.org/images/49973/.

The FMRI experimental dataset was used to generate realistic data for the simulation assessments. We simulated time-series data matching the data size, spectra and covariance matrix of the FMRI experimental dataset.

## Results

### Model validation and simulations

#### Basic simulations

**Fig. 4:**
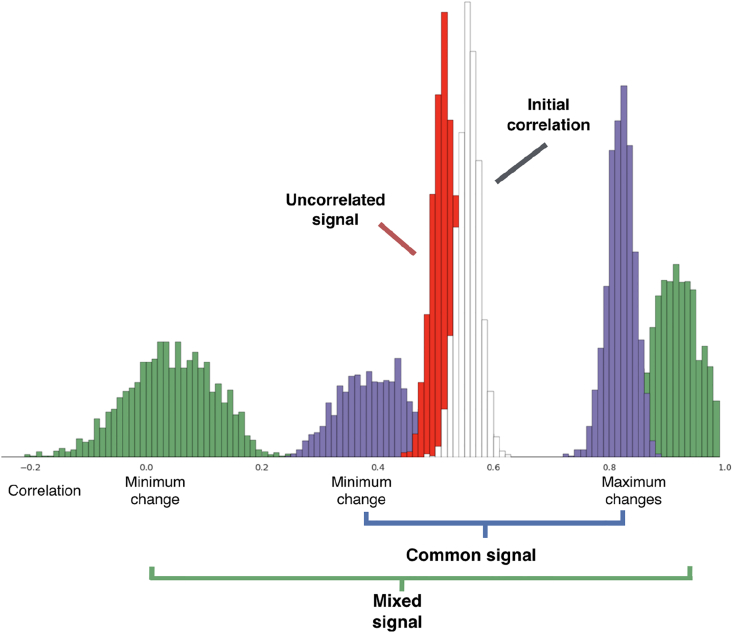
The effects on correlation of additive signals producing a 20% variance change. The plot reflects a scenario where both regions increase in variance by 20%, with an initial correlation of 0.58. The white histogram reflects the distribution of observed correlations in state *A*. The red histogram represents the expected distribution of correlation in state *B*, if the observed 20% change in variance was associated with uncorrelated signals. The blue histograms reflect the distributions of maximum and minimum changes in correlation if variance changes were due to a common additive signal component. Finally, the green histograms reflect the distributions of maximum and minimum changes in correlation when variance changes are due to any additive additions of signal.

We first demonstrate the types of changes in signal that additive signal can produce using simulations. Fig. 4 shows the effects of additive signal changes on the observed correlation of two nodes with initial correlation of 0.58. The white histogram shows the distribution of the observed correlation in this initial state. We simulated the effects of additions of signal producing 20% increases in the standard deviation of both nodes. The red histogram shows the distribution of observed correlation of the second state, when the additive signal was uncorrelated across nodes. On average, an addition of uncorrelated signal produced a drop in observed correlation to 0.40, with a 95% range of (0.35, 0.46). Note that our null-hypothesis tests account for uncertainty in the observed covariances, leading to a range of potential observed changes in correlation, if the change were due to uncorrelated signal. Even observations of increases in correlation with increases in variance may not be adequate to reject a null hypothesis of a change in uncorrelated signals. The blue histograms show the increases in observed correlation produced when a common signal component was added to both regions. Here, the extent of increase will depend on the correlation of the new signal, $N_{c}$ to the initial signals. The two histograms correspond to the effects of those common signals that produce the smallest and largest changes in correlation. Common signals producing a smallest change in correlation on average produced an observed correlation of 0.64, with a 95% minima of 0.61. Those common signals producing the largest increase in signal produce an average observed correlation of 0.71, with a 95% maxima of 0.73. Changes produced by the broade $a_{33}$ and $a_{43}$r class of additive signals are represented by the pair of green histograms. This class can account for a wider range of changes in correlation associated with the 20% increase in standard deviation. Here, the minimum correlation is distributed around 0.02, while the mean maximum correlation is 0.86, with an overall 95% interval of (−0.09, 0.89). Mixtures of additive signals may produce correlations lower than that produced by increases in uncorrelated signal as the signals added to the two nodes can be negatively correlated with each other. Supporting results show how initial correlation alters the effects of different additive signals (Supp Fig. 1).

#### Inference on simulated network changes

**Fig. 5:**
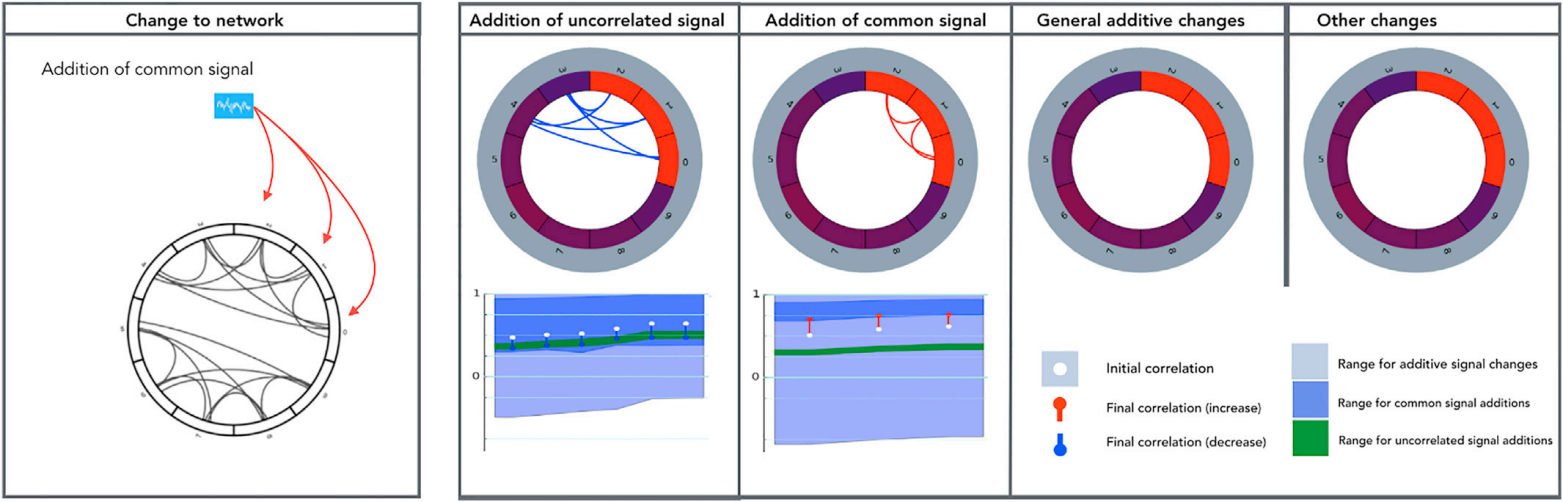
Example analysis of simulated changes in brain networks. Grey connections in the first column indicate nodes with a positive correlation (between 0.3 and 0.7) in the initial state, and red arrows point into nodes to indicate that additional signal was injected in the second state, producing an increase in variance of 20%. Here, a common stochastic process was added to nodes 1–3, uncorrelated with existing signal. The remaining columns represent connections that were detected as showing significant changes in correlation across states, FDR corrected (*α* = 0.2). **Upper row** Each circle plot represents connections determined to fall within a particular class of ASC change. Red connections indicate connections showing increases in correlation after the injection of signal, blue decreases. Node colors similarly represent change in variance. Note that, for clarity, connections falling within subclasses are not included in the more general ASC classes. The final (here, empty) column shows connections that cannot be explained by additive changes. **Lower row** The lower plots represent the distribution of absolute correlation changes for the shown connections. Note that it is not intended to be possible to identify specific connections. White dot - correlation in initial state. Blue/red dot - correlation in second state. Colour fill - range of correlation in second state that could be explained by the additive signal change class. The decreases in correlation between nodes are identified as potentially indicative of increases in uncorrelated signal, but could also be explained by common or other additive changes. The increased correlations between the three nodes receiving the introduced signal are identified as potentially indicative of increases in a common signal (but not a change in uncorrelated signal).

Fig. 5 shows results from an analysis of a simulated scenario of the addition of a common signal into certain nodes of a 10 node network, using the Monte Carlo inference procedure. Here, addition of a common signal into three nodes produced 20% changes in variance. The additive analysis procedure correctly identified nodes sharing a common increase in signal. Additionally, it correctly identified decorrelations of these nodes with other nodes not receiving the signal as changes consistent with increases in uncorrelated signal. This occurred only for connections with some initial correlation. The lower plots indicate the specific changes in correlation for those connections showing a change in correlation, and the range of changes that could be explained by ASC. Note that for these relatively large variance changes, a broad range of changes in correlation could be explained by additive changes in signal.

### Analysing functional connectivity changes in experimental FMRI dataset

**Fig. 6:**
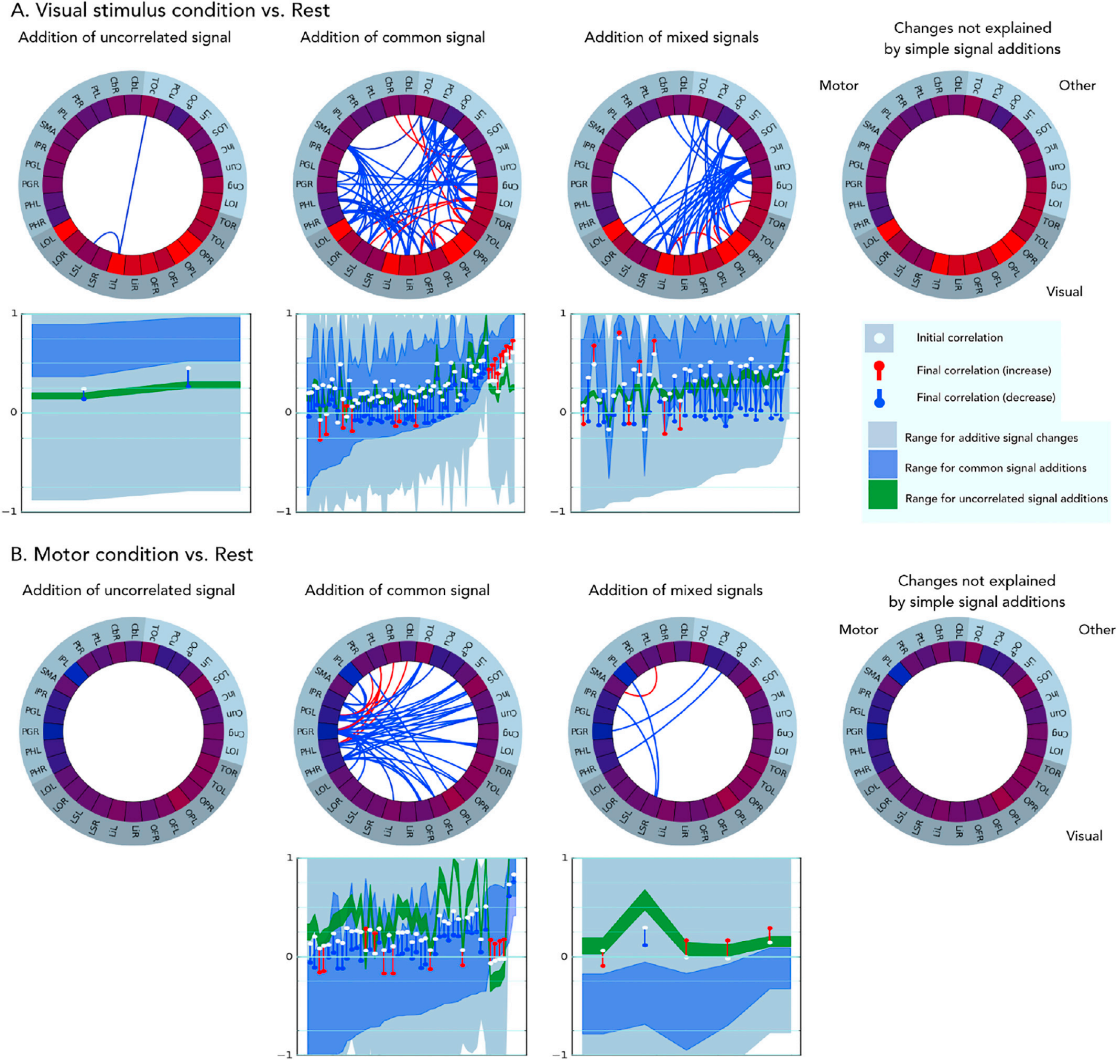
ASC analysis of FC changes between rest and visual stimulation (A), and rest and a motor condition (finger tapping) (B). Plot organisation is described in Fig. 5, region label key is in Table 1 (Supplementary Material). Red connections indicate connections showing increased in correlation in the non-rest conditions, blue decreases. The majority of significant changes in correlation can be explained by additive changes in signal. The visual condition produced increases in variance in visual regions relative to rest, and was associated with increases in correlation between visual nodes that could be explained by increases in common signal or other additive signal changes. Decorrelation with other regions could also be explained by additive changes. The motor condition produced modest reductions in variance in motor regions, which were nevertheless enough for additive changes (common signal) to explain the changes in correlation, including decreases between cortical motor regions, and increases in correlation of cortical motor regions with cerebellum.

We next present an application of the approach to analysing an FMRI study of FC changes across resting and steady-state motor and visual task conditions. Fig. 6 presents the circle-plot results from the additive signal analysis of contrasts of a standard resting state and states in which continuous, visual stimulation (A) and finger tapping (B) was occurring. For clarity, the ROI nodes are restricted to consist of regions relevant to the visual and motor tasks, identified in a prior localisation procedure. Further contrasts involving combined visual and motor conditions are shown in Supp Fig. 2.

Overall, many node-pairs showed differences in correlations across the rest and the steady-state conditions, many of which were lower during the active conditions compared to rest. The majority of these changes could be explained by additions of a single common shared signal (column 2), or other additive signal changes (column 3). The pattern of these changes showed consistencies for states involving the same task types.

The ASC provides considerable insight into the possible nature of the signal changes under lying the different task states. Visual stimulation (Fig. 6A) produced strong increases in variance within visual regions. This was associated with increases in correlation between certain visual brain regions that could be explained by increases in a common shared signal component. A larger number of reductions in correlation could also be explained by the increases in signal in visual regions. Here, this change reduced correlations, possibly indicating that the new signal in the visual regions was weakly correlated with activity in the non-visual regions.

The finger tapping task (Fig. 6B) produced modest reductions in variance in motor-related brain regions, along with a reduction in correlations between these regions and other regions. All of these changes could be explained by additive signal changes (i.e. additional common or partly shared signal in the rest state compared to finger tapping). Many connections were able to be explained purely by reductions in a common signal. In many cases, correlation reduced from around 0.2 to close to 0. The motor cortical regions also showed some cases of increases in correlation with specific brain regions associated with motor control, namely putamen and cerebellum. The changes involving the cerebellum could not be explained by additive changes, indicating a synchronisation of cerebellar regions with motor regions with limited change in the amplitude of fluctuations in the regions.

The combined visual-motor condition (Supp Fig. 2A) produced a pattern of extensive changes in FC which had correspondences to the changes seen in the two single-task conditions: visual and motor cortical regions showing similar increases and decreases in variance. The final condition, which required subjects to attend to specific changes in the movie (Supp Fig. 2B), produced more extensive reductions in signal variance, particularly in default mode regions. However, increases in variance in visual regions were lower. It is possible that the broad reductions in variance across the brain in this condition counteracted local visual-stimulus related increases in variance. Increases in correlations between some motor cortical regions and the right cerebellum, which were near zero at rest, could not be explained by additive changes in signal.

## Discussion

Used on its own, correlation provides an ambiguous characterization of how the relationships between signals change (Friston, 2011, Cole et al., 2016). For example, a change in correlation could be produced by increases in common signal between nodes, decreases in uncorrelated signal, or more complex changes in activity. More complex generative modelling approaches can potentially disambiguate changes in connectivity, but require underlying signal components to be well defined a-priori. Some disambiguation can be provided by assessing the covariance matrix directly (Cole et al., 2016). Assessing the covariance matrices is useful, as it utilises the same summary measures used to produce the correlation measure, adding no further inputs to the analysis. However, this approach does not provide insight into whether observed variance changes are sufficient to suggest particular types of change in the signal underlying the change in correlation. The definition of classes of Additive Signal Change (ASC) permits explicit hypothesis testing of the covariance matrix with minimal additional assumptions. ASC analysis is fundamentally an approach to making inferences from the relationship of changes of the diagonal of the covariance matrix (variances) and changes of the off-diagonal (covariances/correlations). It defines a natural class of changes that are likely to occur in many scenarios. For example, many systems will include largely independent signal sources that may change in amplitude across states. The class excludes changes where existing signal is replaced by signal with a different relationship to other nodes. We have described important subclasses of ASC, and have described methods for null-hypothesis testing for which of the ASC classes can potentially explain observed data.

The ASC classes are not linked to specific models of underlying functional activity, but can guide interpretation, particularly when multiple connections are assessed together. For example, consider an ASC analysis comparing a patient group to control. If the analysis suggests that reduced correlation between a set of functionally related nodes in the patient group may have been produced by reductions in a common signal, this may suggest that this network is less co-active with a particular state. Alternatively, if the reduced correlation was instead found be explained by reductions in uncorrelated signals, associated with greater variance seen across the entire brain, the investigator may look towards global physiological factors or motion artefacts. The class of ASC incorporates a broader range of related scenarios where signals are partially correlated across nodes. Such signals may occur in many contexts. For example, the time course of brain activity associated with a particular cognitive task will not perfectly coincide across brain regions with different roles in the task. Similarly, the effects of head motion on signal will typically vary across brain regions. The ASC analysis will be useful for identifying when FC changes can be explained by such scenarios, rather than more complex changes in signal properties. ASC analysis can guide the fitting of more complex models of brain activity, ensuring that important features of signal variability are not overlooked.

Our results on FMRI data suggest that many changes in FMRI correlation can be explained by additive changes in signal. This has important implications for the interpretation of FC analyses, where changes in correlation are often broadly interpreted as changes in neural coupling, with limited consideration of the specific nature of the underlying changes in signal properties. A majority of the observed changes in correlation were associated with changes in variance in one or both nodes that were substantial enough to explain the correlation changes in terms of additive signal changes. For example, the increases in correlation between visual regions during visual stimulation could be explained by an addition of common signal corresponding to the observed increases in variance. These putative additions of signal could also explain the decorrelations between visual regions and regions that did not show a similar increase in variance.

The ASC analysis provided insight into the striking differences in effects of motor and visual tasks on FC, where these tasks had opposite effects on the variance of activity in activated brain networks. Changes in measured FC will be sensitive to differences in the stability and amplitude of activity across states, which may vary for a variety of reasons. The ASC analysis identified some cases where changes in correlation could not be explained by additive changes in signal. For example, the motor task produced an increase in correlation between cerebellum and SMA with little change in the variances of either region. Rejection of the ASC null hypothesis suggests that simple additive signal changes cannot explain the observed changes. In the ASC framework, this corresponds to scenarios where the new signal is negatively correlated with the existing signal (i.e. new signal replaces existing signal).

Detailed understanding of the underlying dynamics is not possible from an analysis of covariances. However, additional insight can be gained from reviewing ASC results across a variety of connections and states. For example, the visuo-motor attention task appeared to broadly dampen fluctuations, including in regions where visual stimulation had otherwise increased the amplitude of fluctuations. Visual regions showed correspondingly smaller increases in correlation. Changes induced by head-motion will typically be additive, and show particular spatial patterns (Van Dijk et al., 2012). These results make it clear that correlation changes on their own may provide limited insight into the complex array of changes in underlying changes in FC. Evidence of changes in intrinsic coupling and decoupling between nodes can be detected, but must be carefully distinguished from changes that might be explained by additive changes in the amplitude of signal components.

Similar challenges for interpretation apply when covariance or regression co-efficients are studied in isolation. In FMRI, functional connectivity analysis approaches such as psycho-physiological interactions (PPI) (Friston et al., 1997, O'Reilly et al., 2012) and dual regression (Filippini et al., 2009) estimate group level statistics of regression coefficients of signals derived from preselected nodes or networks applied to individual voxels. The sources for observed changes in these analyses remain ambiguous unless variance is explicitly characterized. ASC analysis could be used in a seed-based mapping analysis, identifying all voxels showing a particular class of correlation change with a given seed. Decoding approaches utilizing functional connectivity features are particularly sensitive to the effects of confounds, and will benefit from an ability to better characterize the nature of signal changes driving the predictions from FC changes (Richiardi et al., 2011, Duff et al., 2013, Demertzi et al., 2015). The approach described here has analogues in the frequency domain, where additive changes signal components predictably alter coherence (Cole et al., 2016).

While the results presented here reflect modulations due to continuous induced task-driven activity (Shirer et al., 2012, Zöller et al., 2017), the methods and conclusions are relevant to studies of pure rest states, such as differences across patient groups, or assessment of individual differences. Along with the many studies reporting differences in resting state FC across different subject groups, numerous studies have reported differences in the amplitude of fluctuations (Duff et al., 2008, Zou et al., 2008, Yan et al., 2013). However, variance changes may be more challenging to track in multi-session and parallel group studies, as there will be additional session-to-session and subject-to-subject sources of uncontrolled variability. Careful denoising and normalisation of signal amplitudes will increase sensitivity.

The ASC analysis will provide a useful guide for designing and interpreting effective connectivity modelling strategies such as DCM (Stephan et al., 2007, Friston, 2011) where model estimation will be strongly influenced by the covariance structure. In neuroscience, causal modelling approaches are often focused on characterizing the structure of directed connections within smaller sized networks. In this context, additive signals could be produced by increases in incoming connections to a node, or indirect effects associated with changes in connectivities of nodes with indirect connectivity to the node of interest. A prior ASC analysis could be used to identify patterns of functional connectivity likely to influence causal modelling, and guide ROI selection and other model choices. When interpreting causal model fits it will be useful to cross-check identified changes in coupling with their implied additions of signal to specific nodes. ASC mapping analysis could also be used to map regions not included in the causal model that show similar overall patterns of covariance change, which will indicate the spatial distribution of brain activity that may be associated with a particular causal role.

As an approach that enhances FC analyses, ASC also inherits some of their limitations. Like FC analyses (and related covariance-based analyses such as PPI), ASC assumes that the data at each time point can be well represented using a multivariate Gaussian distribution whose mean is zero, and whose covariance matrix is assumed stationary over time (Lund et al., 2006, Handwerker et al., 2012, Zalesky et al., 2014). For FMRI, the extensive autocorrelation means long scan durations are important: recent studies suggest scan lengths of 13 min substantially improves FC stability (Birn et al., 2013). This means that for shorter duration scans it can be difficult to reject the null hypothesis that a change was due to an Additive Change, even when observed changes in variance are small. The inference procedure implemented in ASC also relies on Gaussianity assumptions, using the approximation that the covariance matrices follow Wishart distributions. Like standard FC analysis, ASC will be sensitive to global signal variations and confounds. However, it can be useful to detect these effects (e.g. identifying possible changes due to uncorrelated noise).

While we focus here on temporally stationary processes, we expect the Additive Signal formulation to be a valuable tool for the analysis of dynamic functional connectivity, where associations between correlation and variance will also exist (Handwerker et al., 2012). One application ASC could be with approaches that model dynamic FC as the stochastic switching between different covariance states, where ASC could be used to interpret transitions between states. Approaches that model dynamic FC using Hidden Markov Models actually identify brain states based on changes over time in either variance or correlation, by explicitly modelling state-dependent covariance matrices (Hansen et al., 2015). For example, approaches that model dynamic FC using Hidden Markov Models actually identify brain states based on changes over time in either variance or correlation, by explicitly modelling state-dependent covariance matrices (Baker et al., 2014, Vidaurre et al., 2017b, Vidaurre et al., 2017a). However, these approaches do not determine whether the variance changes are adequate to explain the change in correlation, and so would be enhanced by the use of ASC (applied to help interpret differences between the different (applied to help interpret differences between the different estimated states).

A number of improvements and extensions could be made to the ASC protocol. The present analyses is either performed on individual data samples or concatenates across samples (e.g. subjects). It may be possible to devise a random effects or other hierarchical modelling approach to ASC that provides a useful characterization of changes at a group level while accounting for variation across subjects (Woolrich et al., 2004, Kasess et al., 2010). Our FDR-based thresholding approach may not be optimal for identifying the extent of connections reliably changing in FC. Many of our putative additive changes would produce a clustering of nodes with altered FC - for example an increase in a signal component common to various nodes across a network. Network-specific correction methods accounting for such structure are likely to provide more sensitive detection of differences across conditions (Zalesky et al., 2010).

ASC is straightforward to apply to partial correlation (partial covariance) matrices. Here the analysis will ignore variance that can be accounted for by other nodes: putative common signals will reflect variance unique to the pair of regions under investigation, while uncorrelated signal will be reflect signal unique to one node. As for any partial correlation analysis, each pairwise analysis would be dependent on the overall set of regions assessed, and the nature of regularisation employed. Investigations must take into account both full and partial correlations to ensure that common signals partialled from pairs of nodes are characterized. Signals common signals Related extensions would be to extend the model to characterize three or more regions simultaneously, identifying signals that are common across subsets of regions. Another strategy to provide finer dissection of variance components could be to simultaneously model multiple states simultaneously.

We have presented an additive signal change model linking correlation and variance that can substantially enhance the description of functional connectivity. The additional information provided by classifying correlation changes into those that can be explained by specific simple changes in signal, and those that are suggestive of coupling changes, provides a less ambiguous characterization of functional connectivity that may make it easier to share and assess studies of functional and effective connectivity.

## Competing interests

No competing interests.

## Code and data availability

Source code for performing these analyses is available for download from the FSL website. www.fmrib.ox.ac.uk/fsl (git.fmrib.ox.ac.uk/eduff/ampconn). Source data is being submitted to Neurovault: www.neurovault.org.
