# Supplementary material for: Disambiguating brain functional connectivity
Source: Neuroimage. 2018 Jun;173:540–50. doi: 10.1016/j.neuroimage.2018.01.053 (PMC5929905; doi:10.1016/j.neuroimage.2018.01.053)
Supplement: Supplementary materials [file mmc1.pdf]

Supplementary Material: Disambiguating brain functional connectivity

1. Methods

1.1. Model

We consider nodes X and Y, which can be observed in multiple states. In each state the nodes produce signals distributed according to an ergodic stochastic process. Consider a state A, where the signals have distributions $X_A(t)$ and $Y_A(t)$:

$$X_A(t) = \{X_t, t \in T\}$$
$$Y_A(t) = \{Y_t, t \in T\}$$

T specifies some experimental observational period. These periods enable the estimation of covariances:

$$\Sigma_{X_A,Y_A} = \begin{bmatrix} \sigma^2_{X_A} & \sigma_{X_A}\sigma_{Y_A}\rho_{X_AY_A} \\ \sigma_{X_A}\sigma_{Y_A}\rho_{X_AY_A} & \sigma^2_{Y_A} \end{bmatrix}$$

Additive signal change analysis explores the putative relationship between two states, say A and B. State B is modelled by an introduction of new signals to A, X_N and Y_N, such that:

$$X_B = X_A + X_N$$
$$Y_B = Y_A + Y_N$$

The addition of X_N and Y_N and will alter covariances. We can consider the overall covariance structure of putative signals:

$$\left\{ \begin{array}{c} X_A \\ X_N \\ Y_A \\ Y_N \end{array} \right\} \sim M \left\{ 0, \begin{array}{cccc} \sigma^2_{X_A} & \sigma_{X_A}\sigma_{X_N}\rho_{X_A,X_N} & \sigma_{X_A}\sigma_{Y_N}\rho_{X_A,Y_A} & \sigma_{X_A}\sigma_{Y_A}\rho_{X_A,Y_N} \\ \sigma_{X_A}\sigma_{X_N}\rho_{X_A,X_N} & \sigma^2_{X_N} & \sigma_{X_N}\sigma_{Y_N}\rho_{X_N,Y_A} & \sigma_{X_N}\sigma_{Y_A}\rho_{X_N,Y_N} \\ \sigma_{X_A}\sigma_{Y_A}\rho_{X_A,Y_A} & \sigma_{X_N}\sigma_{Y_A}\rho_{Y_A,X_N} & \sigma^2_{Y_A} & \sigma_{Y_N}\sigma_{Y_A}\rho_{Y_A,Y_N} \\ \sigma_{X_A}\sigma_{Y_N}\rho_{X_A,Y_N} & \sigma_{X_N}\sigma_{Y_N}\rho_{X_N,Y_N} & \sigma_{Y_A}\sigma_{Y_N}\rho_{Y_A,Y_N} & \sigma^2_{Y_N} \end{array} \right\} \quad (1)$$

We are interested in making inferences regarding the nature of X_N and Y_N from available data. For example, whether they could be uncorrelated signals (e.g. uncorrelated noise), or identical signals (e.g. a common signal component). This involves making inferences relating to variables in Eqn. (1), based on observed covariances (Q_A, Q_B). To make inferences on these variables, we determine whether observed functional connectivity changes can be explained by changes when specific constraints on these variables hold. We pose this as a problem of determining whether the observed covariance in state B, Q_B (equally, the corresponding correlation) falls within the limits implied by the specified constraints given Q_A and the observed changes in variance (e.g. $\hat{\sigma}_{X_B} - \hat{\sigma}_{X_A}$).

Identification of limits on σ_{X_B,Y_B} given equality and inequality constraints can be achieved by solving the Karush-Kuhn-Tucker (KKT) conditions. We identify limits for three classes of change: additive signal changes, changes in a common signal, and changes in uncorrelated signal. Each of these correspond to specific constraints on the covariance structure. We first describe results without considering uncertainty due to limited data sampling. We then describe an approach to account for uncertainty.

1.1.1. Bounds on correlation changes produced by Additive Signal Changes.

We define Additive Signal Changes as changes produced by the addition of new signal to a node that has non-negative correlation with the existing signal. Such changes always produce a change in variance, and encompass common types of signal changes across many different contexts. We aim to determine whether observed changes in σ_{X_B,Y_B} could be explained by such changes. The additive signal may appear either of the two states, for either node. Using the above formulation, we have:

$$X_B = X_A + X_N, \text{where,} \begin{cases} \rho_{X_A,X_N} \geq 0 & \text{if } \sigma^2_{X_B} \geq \sigma^2_{X_A} \\ \rho_{X_B,-X_N} \geq 0 & \text{otherwise.} \end{cases}$$

$$Y_B = Y_A + Y_N, \text{where,} \begin{cases} \rho_{Y_A,Y_N} \geq 0 & \text{if } \sigma^2_{Y_B} \geq \sigma^2_{Y_A} \\ \rho_{Y_B,-Y_N} \geq 0 & \text{otherwise.} \end{cases}$$

For an uncorrelated additive signal the difference in variance between the original and new signal will be equal to the variance of the additional signal. For a positively correlated additive signal the variance change will always exceed the variance of the signal being added. This implies, from above:

$$\sigma^2_{X_N} \leq |\sigma^2_{X_B} - \sigma^2_{X_A}| \tag{2}$$

$$\sigma^2_{Y_N} \leq |\sigma^2_{Y_B} - \sigma^2_{Y_A}| \tag{3}$$

To simplify the analysis, we analyse the system in terms of orthogonal component unit vectors. We define the first component, S_1, to correspond to the process X_A. The second component, S_2, corresponds to that component of Y_A that is orthogonal to X_A ($O^{Y_A}_{X_A}$). The next component S_3 corresponds to the signal of X_N orthogonal to the previous two components, and the final component S_4 corresponds to remaining variability in Y_N that is orthogonal to other signals. Thus the putative signals can be defined:

$$\begin{aligned} X_A &= a_{11}S_1 \\ Y_A &= a_{21}S_1 + a_{22}S_2 \\ X_N &= a_{31}S_1 + a_{32}S_2 + a_{33}S_3 \\ Y_N &= a_{41}S_1 + a_{42}S_2 + a_{43}S_3 + a_{44}S_4 \end{aligned}$$

From observations of original covariance and variances (σ_{X_A,Y_A}), we can estimate a_{11}, a_{21}, and a_{22}. We therefore have unknowns a_{31}, a_{32}, a_{33}, a_{41}, a_{42} a_{43}, and a_{44}.

We can define σ_{X_B,Y_B} in terms of these components:

$$\begin{aligned} \sigma_{X_B,Y_B} &= \sigma_{X_A+X_N,Y_A+Y_N} \\ &= \sigma_{X_A,Y_A} + \sigma_{X_A,Y_N} + \sigma_{Y_A,X_N} + \sigma_{X_N,Y_N} \\ &= a_{11}a_{21} + a_{11}a_{41} + (a_{21}a_{31} + a_{22}a_{32}) + (a_{31}a_{41} + a_{32}a_{42} + a_{33}a_{43}) \end{aligned}$$

We can define equality constraints associated with the overall variances of signals observed in states A and B:

$$\begin{aligned} \text{con}(\sigma^2_{X_B}) &= a^2_{31} + a^2_{32} + a^2_{33} + 2a_{11}a_{31} - (\sigma^2_{X_B} - \sigma^2_{X_A}) = 0 \\ \text{con}(\sigma^2_{Y_B}) &= a^2_{41} + a^2_{42} + a^2_{43} + a^2_{44} + 2a_{21}a_{41} + 2a_{22}a_{42} - (\sigma^2_{Y_B} - \sigma^2_{Y_A}) = 0 \end{aligned}$$

And inequality constraints associated with the requirement that changes are additive, as defined in Eqns. (2) & (3):

$$\begin{aligned} \text{con}(add_X) &= a^2_{31} + a^2_{32} + a^2_{33} - |\sigma^2_{X_B} - \sigma^2_{X_A}| \leq 0 \\ \text{con}(add_Y) &= a^2_{41} + a^2_{42} + a^2_{43} + a^2_{44} - |\sigma^2_{Y_B} - \sigma^2_{Y_A}| \leq 0 \end{aligned}$$

To simplify derivations, the latter two constraints are converted to:

$$\begin{aligned} \text{con}(add_X) &= (\sigma^2_{X_B} - \sigma^2_{X_A}) - |\sigma^2_{X_B} - \sigma^2_{X_A}| - 2a_{11}a_{31} \leq 0 \\ \text{con}(add_Y) &= (\sigma^2_{Y_B} - \sigma^2_{Y_A}) - |\sigma^2_{Y_B} - \sigma^2_{Y_A}| - 2a_{21}a_{41} - 2a_{22}a_{42} \leq 0 \end{aligned}$$

The Langrangian for the maximum covariance is then given by:

$$\begin{aligned} \mathcal{L}(a,\lambda) = {} & a_{11}a_{21} + a_{11}a_{41} + (a_{21}a_{31} + a_{22}a_{32}) + (a_{31}a_{41} + a_{32}a_{42} + a_{33}a_{43}) \\ & - \lambda_{\sigma^2_{X_B}}\left(a^2_{31} + a^2_{32} + a^2_{33} + 2a_{11}a_{31} - (\sigma^2_{X_B} - \sigma^2_{X_A})\right) \\ & - \lambda_{\sigma^2_{Y_B}}\left(a^2_{41} + a^2_{42} + a^2_{43} + a^2_{44} + 2a_{21}a_{41} + 2a_{22}a_{42} - (\sigma^2_{Y_B} - \sigma^2_{Y_A})\right) \\ & - \lambda_{add_X}\left((\sigma^2_{X_B} - \sigma^2_{X_A}) - |\sigma^2_{X_B} - \sigma^2_{X_A}| - 2a_{11}a_{31}\right) \\ & - \lambda_{add_Y}\left((\sigma^2_{Y_B} - \sigma^2_{Y_A}) - |\sigma^2_{Y_B} - \sigma^2_{Y_A}| - 2a_{21}a_{41} - 2a_{22}a_{42}\right) \end{aligned}$$

The KKT points have to satisfy:

$$\frac{\partial \mathcal{L}}{\partial a_{31}} = a_{21} + a_{41} - 2\lambda_{\sigma^2_{X_B}}(a_{11} + a_{31}) + 2\lambda_{add_X} a_{11} = 0$$
$$\frac{\partial \mathcal{L}}{\partial a_{32}} = a_{22} + a_{42} - 2\lambda_{\sigma^2_{X_B}} a_{32} = 0$$
$$\frac{\partial \mathcal{L}}{\partial a_{33}} = a_{43} - 2\lambda_{\sigma^2_{X_B}} a_{33} = 0$$
$$\frac{\partial \mathcal{L}}{\partial a_{41}} = a_{11} + a_{31} - 2\lambda_{\sigma^2_{Y_B}}(a_{21} + a_{41}) + 2\lambda_{add_Y} a_{21} = 0$$
$$\frac{\partial \mathcal{L}}{\partial a_{42}} = a_{32} - 2\lambda_{\sigma^2_{Y_B}}(a_{22} + a_{42}) + 2\lambda_{add_Y} a_{22} = 0$$
$$\frac{\partial \mathcal{L}}{\partial a_{43}} = a_{33} - 2\lambda_{\sigma^2_{Y_B}} a_{43} = 0$$
$$\frac{\partial \mathcal{L}}{\partial a_{44}} = 2\lambda_{\sigma^2_{Y_B}} a_{44} = 0$$

For the inequality constraints:

$$\lambda_{add_X}\Big((\sigma^2_{X_B} - \sigma^2_{X_A}) - |\sigma^2_{X_B} - \sigma^2_{X_A}| - 2a_{11}a_{31}\Big) = 0$$
$$\lambda_{add_Y}\Big((\sigma^2_{Y_B} - \sigma^2_{Y_A}) - |\sigma^2_{Y_B} - \sigma^2_{Y_A}| - 2a_{21}a_{41} - 2a_{22}a_{42}\Big) = 0$$
$$\lambda_{add_X}, \lambda_{add_Y}, \lambda_{\sigma^2_{Y_B}} \geq 0$$

With equality constraints:

$$a_{31}^2 + a_{32}^2 + a_{33}^2 + 2a_{11}a_{31} - (\sigma^2_{X_B} - \sigma^2_{X_A}) = 0$$
$$a_{41}^2 + a_{42}^2 + a_{43}^2 + a_{44}^2 + 2a_{21}a_{41} + 2a_{22}a_{42} - (\sigma^2_{Y_B} - \sigma^2_{Y_A}) = 0$$

To identify KKT points, it is necessary to assess cases where different numbers of inequality constraints are active.

Case 1: No active constraints (i.e.$\lambda_{add_X}, \lambda_{add_Y} = 0$) We have:

$$a_{21} + a_{41} - 2\lambda_{\sigma^2_{X_B}}(a_{11} + a_{31}) = 0$$
$$a_{22} + a_{42} - 2\lambda_{\sigma^2_{X_B}} a_{32} = 0$$
$$a_{43} - 2\lambda_{\sigma^2_{X_B}} a_{33} = 0$$
$$a_{11} + a_{31} - 2\lambda_{\sigma^2_{Y_B}}(a_{21} + a_{41}) = 0$$
$$a_{32} - 2\lambda_{\sigma^2_{Y_B}}(a_{22} + a_{42}) = 0$$
$$\frac{\partial \mathcal{L}}{\partial a_{43}} = a_{33} - 2\lambda_{\sigma^2_{Y_B}} a_{43} = 0$$
$$\frac{\partial \mathcal{L}}{\partial a_{44}} = 2\lambda_{\sigma^2_{Y_B}} a_{44} = 0$$

Dividing out $\lambda_{\sigma^2_{Y_B}}$, we obtain:

$$2\lambda_{\sigma^2_{Y_B}} = \frac{a_{11} + a_{31}}{a_{21} + a_{41}} = \frac{a_{32}}{a_{22} + a_{42}} = \frac{a_{33}}{a_{43}} \tag{4}$$

With $a_{44} = 0$. This implies $\rho_{X_B,Y_B} = \pm 1$. This solution will only be feasible if the variance changes in both regions are adequate to equalise ratios. The calculation in the all-active constraints case below identifies when variance is adequate for perfect correlation. When variance changes are adequate, additional variance can be accounted for by appropriate increases in a_{33} and a_{43} to maintain the above ratios.

Case 2: All active constraints

Here we have:

$$a_{21} + a_{41} - 2\lambda_{\sigma^2_{X_B}}(a_{11} + a_{31}) + 2\lambda_{add_X} a_{11} = 0 \tag{5}$$
$$a_{22} + a_{42} - 2\lambda_{\sigma^2_{X_B}} a_{32} = 0 \tag{6}$$
$$a_{43} - 2\lambda_{\sigma^2_{X_B}} a_{33} = 0 \tag{7}$$
$$a_{11} + a_{31} - 2\lambda_{\sigma^2_{Y_B}}(a_{21} + a_{41}) + 2\lambda_{add_Y} a_{21} = 0 \tag{8}$$
$$a_{32} - 2\lambda_{\sigma^2_{Y_B}}(a_{22} + a_{42}) + 2\lambda_{add_Y} a_{22} = 0 \tag{9}$$
$$a_{33} - 2\lambda_{\sigma^2_{Y_B}} a_{43} = 0 \tag{10}$$
$$2\lambda_{\sigma^2_{Y_B}} a_{44} = 0$$

Combining Eqns. (7) and (10) suggests that $\sigma^2_{\lambda_{Y_B}} = \frac{1}{\sigma^2_{\lambda_{X_B}}}$. Combining this with Eqns. (5) and (8), and (6) and (9), we find $2\lambda_{add_Y} = 0$ and $2\lambda_{add_X} = 0$. This produces the same formulation as case 1, which only applies with appropriate variance change.

An alternative scenario satisfying Eqns. (7) and (10) has a_{33}, a_{43}, and a_{44} equal to zero. This restricts solutions to scenarios where the additive signals do not include any signal uncorrelated with existing signals. These solutions can produce the maximum possible correlation change, by adding (or subtracting) signal associated with the other node. The inequality constraints become:

$$2a_{11}a_{31} = (\sigma^2_{X_B} - \sigma^2_{X_A}) - |\sigma^2_{X_B} - \sigma^2_{X_A}| \tag{11}$$
$$2a_{21}a_{41} + 2a_{22}a_{42} = (\sigma^2_{Y_B} - \sigma^2_{Y_A}) - |\sigma^2_{Y_B} - \sigma^2_{Y_A}| \tag{12}$$

We assess cases when variances a greater in state A or B separately.

Variance is greater in state B. Here, the right hand side of these constraints is zero. This implies that we have X_N is uncorrelated with X_A ($a_{31} = 0$), and Y_N uncorrelated with Y_A ($a_{21}a_{41} + a_{22}a_{42} = 0$). With the these results, we have:

$$a^2_{32} = \sigma^2_{X_B} - \sigma^2_{X_A}$$
$$a^2_{41} + a^2_{42} = \sigma^2_{Y_B} - \sigma^2_{Y_A}$$

Eqn. (12), with the right side equal to 0 shows $a_{42} = -a_{41}\frac{a_{21}}{a_{22}}$, we therefore have:

$$a^2_{41}(1 - \frac{a^2_{21}}{a^2_{22}}) = \sigma^2_{Y_B} - \sigma^2_{Y_A}$$
$$a^2_{41} = \frac{a^2_{22}}{a^2_{22} - a^2_{21}}(\sigma^2_{Y_B} - \sigma^2_{Y_A})$$

These results permit the calculation of maximum covariance and correlation:

$$\sigma_{X_B,Y_B} = a_{11}a_{21} + a_{11}a_{41} + a_{22}a_{32} + a_{32}a_{42}$$

This solution will identify maximum (minimum) correlation if the variance changes are not adequate to achieve maximum correlation of 1 (-1) (Case 1). It is necessary to determine if the variance changes are adequate to achieve maximum correlation, as in these cases present solution provides non-optimal solutions. We can do this by assessing the ratios examined in Eqn. (4) for Case 1. If variance changes are not enough for Eqn. (4) to hold (i.e. a_{41} and a_{32} are small), and we have, instead of the equality of Eqn. (4):

$$\frac{a^2_{11}}{(a_{21} + a_{41})^2} \geq \frac{a^2_{32}}{a^2_{22}}$$

If this equality holds, Case 2 identifies the maximum possible correlation. We may also find that:

$$\frac{a^2_{11}}{(a_{21} + a_{41})^2} \leq \frac{a^2_{32}}{a^2_{22}}$$

In this scenario, a correlation of 1 can be achieved with smaller values of a_{41} and a_{32}, offset by appropriate addition of other signal components (a_{31}, a_{42}) to achieve the observed variance changes to achieve Eqn. (4) (Case 1).

Variance is greater in state A. In this scenario we have, from Eqns. (11) and (11):

$$2a_{11}a_{31} = 2(\sigma^2_{X_B} - \sigma^2_{X_A})$$
$$2a_{21}a_{41} + 2a_{22}a_{42} = 2(\sigma^2_{Y_B} - \sigma^2_{Y_A})$$

These equalities imply that X_N is uncorrelated with X_B, analogous to above:

$$
\begin{aligned}
\sigma_{X_B,X_N} &= a_{11}a_{31} + a_{31}^2 + a_{32}^2 + a_{33}^2 \\
&= a_{11}a_{31} + a_{31}^2 + a_{32}^2 + a_{33}^2 \\
&= (\sigma_{X_B}^2 - \sigma_{X_A}^2) - (\sigma_{X_B}^2 - \sigma_{X_A}^2) \\
&= 0
\end{aligned}
$$

Similar results show Y_N is uncorrelated with Y_B. We can then obtain expressions for required terms for calculating σ_{X_B,Y_B}:

$$a_{31} = \frac{\sigma_{X_B}^2 - \sigma_{X_A}^2}{a_{11}}$$

a_{33} can be shown to be 0 as for the previous scenario. a_{32} can then be calculated:

$$
\begin{aligned}
a_{31}^2 + a_{32}^2 + a_{33}^2 + 2a_{11}a_{31} &= \sigma_{X_B}^2 - \sigma_{X_A}^2 \\
a_{32}^2 &= \sigma_{X_A}^2 - \sigma_{X_B}^2 - a_{31}^2
\end{aligned}
$$

Similarly, for a_{41} and a_{42}:

$$
\begin{aligned}
2a_{21}a_{41} + 2a_{22}a_{42} &= 2(\sigma_{Y_B}^2 - \sigma_{Y_A}^2) \\
a_{42} &= \frac{\sigma_{Y_B}^2 - \sigma_{Y_A}^2 - a_{21}a_{41}}{a_{22}}
\end{aligned}
$$

Calculating an expression for a_{41}:

$$
\begin{aligned}
a_{41}^2 + a_{42}^2 + 2a_{21}a_{41} + 2a_{22}a_{42} &= \sigma_{Y_B}^2 - \sigma_{Y_A}^2 \\
a_{41}^2 + a_{42}^2 + 2\sigma_{Y_B}^2 - 2\sigma_{Y_A}^2 &= \sigma_{Y_B}^2 - \sigma_{Y_A}^2 \\
a_{41}^2 + a_{42}^2 &= \sigma_{Y_A}^2 - \sigma_{Y_B}^2 \\
a_{41}^2(1 + \frac{a_{21}^2}{a_{22}^2}) &= \sigma_{Y_A}^2 - \sigma_{Y_B}^2 \\
a_{41}(1 + \frac{a_{21}}{a_{22}}) &= \qquad\qquad \sqrt{\sigma_{Y_A}^2 - \sigma_{Y_B}^2} \\
a_{41} &= \pm\frac{a_{22}}{a_{21} + a_{22}}\sqrt{\sigma_{Y_A}^2 - \sigma_{Y_B}^2}
\end{aligned}
$$

The formulations for increases and decreases across states can be mixed if one node shows an increase in variance in a particular state while the other shows a decrease.

Case 3: One active constraint (e.g. $\lambda_{add_X} = 0, \lambda_{add_Y} \neq 0$)

With $\lambda_{add_X} = 0$ we obtain:

$$
\begin{aligned}
a_{21} + a_{41} - 2\lambda_{\sigma_{X_B}^2}(a_{11} + a_{31}) &= 0 \\
a_{22} + a_{42} - 2\lambda_{\sigma_{X_B}^2}a_{32} &= 0 \\
a_{43} - 2\lambda_{\sigma_{X_B}^2}a_{33} &= 0 \\
a_{11} + a_{31} - 2\lambda_{\sigma_{Y_B}^2}(a_{21} + a_{41}) + 2\lambda_{add_Y}a_{21} &= 0 \\
a_{32} - 2\lambda_{\sigma_{Y_B}^2}(a_{22} + a_{42}) + 2\lambda_{add_Y}a_{22} &= 0 \\
a_{33} - 2\lambda_{\sigma_{Y_B}^2}a_{43} &= 0 \\
2\lambda_{\sigma_{Y_B}^2}a_{44} &= 0
\end{aligned}
$$

As for Case 2, combining Eqns. (7) and (10) indicates that $\sigma_{Y_B}^2 = \frac{1}{\sigma_{X_B}^2}$, leading to the same formulation as case 1, which only applies with appropriate variance change. This implies either $\lambda_{\sigma_{Y_B}^2} = 0$, which obtain the same results as for Case 1 (Eqn. (4)), or $a_{33} = 0$ and $a_{43} = 0$, implying Case 2.

1.1.2. Bounds on correlation changes produced by the addition of a common signal.

To model the range of changes that can be produced by changes in a signal that is common across both regions, we modify the above model to have: $a_{41} = ka_{31}$, $a_{42} = ka_{32}$, and $a_{43} = ka_{33}$, with $sign(\sigma_{X_A,Y_A})k > 0$. This requires the additive signal to

both regions to be perfectly correlated. The sign of k is defined to allow for anti-correlated signals, where the common signals is defined to be of opposite sign in the two regions.

The covariance in state B is now:

$$\sigma_{X_B,Y_B} = a_{11}a_{21} + a_{11}ka_{31} + (a_{21}a_{31} + a_{22}a_{32}) + (ka_{31}^2 + ka_{32}^2 + ka_{33}^2)$$

This also alters the constraints, leading to the KKT equation:

$$\begin{aligned}
\mathcal{L}(a,\lambda) = a_{11}a_{21} + a_{11}ka_{31} + (a_{21}a_{31} + a_{22}a_{32}) + ka_{31}^2 + ka_{32}^2 + ka_{33}^2) \\
- \lambda_{\sigma^2_{X_B}}\Big(a_{31}^2 + a_{32}^2 + a_{33}^2 + 2a_{11}a_{31} - (\sigma^2_{X_B} - \sigma^2_{X_A})\Big) \\
- \lambda_{\sigma^2_{Y_B}}\Big(k^2a_{31}^2 + k^2a_{32}^2 + k^2a_{33}^2 + 2a_{21}ka_{31} + 2a_{22}ka_{32} - (\sigma^2_{Y_B} - \sigma^2_{Y_A})\Big) \\
- \lambda_{add_X}\Big((\sigma^2_{X_B} - \sigma^2_{X_A}) - |\sigma^2_{X_B} - \sigma^2_{X_A}| - 2a_{11}a_{31}\Big) \\
- \lambda_{add_Y}\Big(- 2a_{21}ka_{31} - 2a_{22}ka_{32}\Big)
\end{aligned}$$

With KKT points having to satisfy:

$$\begin{aligned}
\frac{\partial\mathcal{L}}{\partial a_{31}} = a_{11}k + a_{21} + 2ka_{31} - 2\lambda_{\sigma^2_{X_B}}(a_{11} + a_{31}) - 2\lambda_{\sigma^2_{Y_B}}(ka_{21} + k^2a_{31}) + 2\lambda_{add_X}a_{11} + 2k\lambda_{add_Y}a_{21} = 0 \\
\frac{\partial\mathcal{L}}{\partial a_{32}} = a_{22} + 2ka_{32} - 2\lambda_{\sigma^2_{X_B}}a_{32} - 2\lambda_{\sigma^2_{Y_B}}(ka_{22} + k^2a_{32}) + 2k\lambda_{add_Y}a_{22} = 0 \\
\frac{\partial\mathcal{L}}{\partial a_{33}} = 2ka_{33} - 2\lambda_{\sigma^2_{X_B}}a_{33} - 2\lambda_{\sigma^2_{Y_B}}k^2a_{33} = 0 \\
\frac{\partial\mathcal{L}}{\partial k} = a_{11}a_{31} + a_{31}^2 + a_{32}^2 + a_{33}^2 - \lambda_{\sigma^2_{Y_B}}\Big(2ka_{31}^2 + 2ka_{32}^2 + 2ka_{33}^2 + 2a_{21}a_{31} + 2a_{22}a_{32}\Big) \\
+\lambda_{add_Y}\Big(2a_{21}a_{31} + 2a_{22}a_{32}\Big) = 0
\end{aligned}$$

with equality constraints:

$$\begin{aligned}
a_{31}^2 + a_{32}^2 + a_{33}^2 + 2a_{11}a_{31} - (\sigma^2_{X_B} - \sigma^2_{X_A}) = 0 \\
k^2a_{31}^2 + k^2a_{32}^2 + k^2a_{33}^2 + 2a_{21}ka_{31} + 2a_{22}ka_{32} - (\sigma^2_{Y_B} - \sigma^2_{Y_A}) = 0
\end{aligned} \tag{13}$$

and inequality constraints:

$$\begin{aligned}
\lambda_{add_X}\Big((\sigma^2_{X_B} - \sigma^2_{X_A}) - |\sigma^2_{X_B} - \sigma^2_{X_A}| - 2a_{11}a_{31}\Big) = 0 \\
\lambda_{add_Y}\Big((\sigma^2_{Y_B} - \sigma^2_{Y_A}) - |\sigma^2_{Y_B} - \sigma^2_{Y_A}| - 2ka_{21}a_{31} - 2ka_{22}a_{32}\Big) = 0 \\
\lambda_{add_X}, \lambda_{add_Y}, \lambda_{\sigma^2_{Y_B}} \geq 0
\end{aligned} \tag{14}$$

Case 1: No active constraints (e.g.$\lambda_{add_X}, \lambda_{add_Y} = 0$)
We now have:

$$\frac{\partial\mathcal{L}}{\partial a_{31}} = a_{11}k + a_{21} + 2ka_{31} - 2\lambda_{\sigma^2_{X_B}}(a_{11} + a_{31}) - 2\lambda_{\sigma^2_{Y_B}}(ka_{21} + k^2a_{31}) = 0 \tag{15}$$

$$\frac{\partial\mathcal{L}}{\partial a_{32}} = a_{22} + 2ka_{32} - 2\lambda_{\sigma^2_{X_B}}a_{32} - 2\lambda_{\sigma^2_{Y_B}}(ka_{22} + k^2a_{32}) = 0 \tag{16}$$

$$\frac{\partial\mathcal{L}}{\partial a_{33}} = 2ka_{33} - 2\lambda_{\sigma^2_{X_B}}a_{33} - 2\lambda_{\sigma^2_{Y_B}}k^2a_{33} = 0 \tag{17}$$

$$\frac{\partial\mathcal{L}}{\partial k} = a_{11}a_{31} + a_{31}^2 + a_{32}^2 + a_{33}^2 - \lambda_{\sigma^2_{Y_B}}\Big(2ka_{31}^2 + 2ka_{32}^2 + 2ka_{33}^2 + 2a_{21}a_{31} + 2a_{22}a_{32}\Big) = 0$$

Eqn. (17) can be rearranged to:

$$a_{33}(2k - 2\lambda_{\sigma^2_{X_B}} - 2\lambda_{\sigma^2_{Y_B}}k^2) = 0 \tag{18}$$

If $2k - 2\lambda_{\sigma^2_{X_B}} - 2\lambda_{\sigma^2_{Y_B}}k^2 = 0$, then we have from Eqns. (15) and (16):

$$\begin{aligned}
a_{11}k + a_{21} - 2\lambda_{\sigma^2_{X_B}}a_{11} - 2\lambda_{\sigma^2_{Y_B}}ka_{21} = 0 \\
a_{22} - 2\lambda_{\sigma^2_{Y_B}}ka_{22} = 0
\end{aligned}$$

Which implies:

$$\sigma^2_{Y_B} = \frac{1}{2k}$$

Substituting into Eqn. (18), we find:

$$a_{11}a_{31} + \frac{1}{k}(a_{21}a_{31} + a_{22}a_{32}) = 0 \tag{19}$$

Finally, substituting this into the equation for σ_{X_B,Y_B}:

$$\begin{aligned}
\sigma_{X_B,Y_B} &= \sigma_{X_A,Y_A} + a_{11}ka_{31} + (a_{21}a_{31} + a_{22}a_{32}) + (ka^2_{31} + ka^2_{32} + ka^2_{33}) \\
&= \sigma_{X_A,Y_A} + a_{11}ka_{31} + (a_{21}a_{31} + a_{22}a_{32}) + k(\sigma^2_{X_B} - \sigma^2_{X_A} - 2a_{11}ka_{31}) \\
&= \sigma_{X_A,Y_A} + k(\sigma^2_{X_B} - \sigma^2_{X_A})
\end{aligned}$$

k can be calculated by solving the quadratic in (13):

$$\begin{aligned}
0 &= k^2a^2_{31} + k^2a^2_{32} + k^2a^2_{33} + 2a_{21}ka_{31} + 2a_{22}ka_{32} - (\sigma^2_{Y_B} - \sigma^2_{Y_A}) \\
&= k^2((\sigma^2_{X_B} - \sigma^2_{X_A}) - 2a_{11}a_{31}) - k * 2(a_{21}a_{31} + a_{22}a_{32}) - (\sigma^2_{Y_B} - \sigma^2_{Y_A}) \\
&= k^2(\sigma^2_{X_B} - \sigma^2_{X_A}) - k^2(2a_{11}a_{31}) - k * 2(a_{21}a_{31} + a_{22}a_{32}) - (\sigma^2_{Y_B} - \sigma^2_{Y_A}) \\
&= k^2(\sigma^2_{X_B} - \sigma^2_{X_A}) - (\sigma^2_{Y_B} - \sigma^2_{Y_A}) \\
k^2 &= \frac{\sigma^2_{Y_B} - \sigma^2_{Y_A}}{\sigma^2_{X_B} - \sigma^2_{X_A}}
\end{aligned}$$

$$\begin{aligned}
\sigma_{X_B,Y_B} =& \sigma_{X_A,Y_A} + k(\sigma^2_{X_B} - \sigma^2_{X_A}) \\
=& \sigma_{X_A,Y_A} + \sqrt{(\sigma^2_{X_B} - \sigma^2_{X_A})(\sigma^2_{Y_B} - \sigma^2_{Y_A})}
\end{aligned}$$

Alternatively, $a_{33} = 0$. Here it is not possible to identify a unique maximum analytically, but we can express σ_{X_B,Y_B} in terms of a_{31} and identify maximum and minimum values via a search over possible values of this term.

Case 2: Active constraints

When active, both constraints require that the new signal is uncorrelated with the existing signal. This implies $a_{31} = 0$. Eqn. (14) then implies $a_{32} = 0$. As this implies: $a_{11}a_{31} + \frac{1}{k}(a_{21}a_{31} + a_{22}a_{32}) = 0$, (e.g. Eqn. (19)) we obtain the same result as the first scenario in Case 1:

$$\sigma_{X_B,Y_B} = \sigma_{X_A,Y_A} + \sqrt{(\sigma^2_{X_B} - \sigma^2_{X_A})(\sigma^2_{Y_B} - \sigma^2_{Y_A})}$$

Case 3: One active constraint.

Here the common signal is uncorrelated with one of the two initial signals. Taking $a_{31} = 0$, we find:

$$\begin{aligned}
a_{33}(2k - \sigma^2_{X_A}) &= 0 \\
a_{22} + a_{32}(2k - \sigma^2_{X_A}) &= 0
\end{aligned}$$

This requires $a_{33} = 0$, for $a_{22} \neq 0$. We can now directly calculate σ_{X_B,Y_B}:

$$\begin{aligned}
a_{32} &= \sqrt{\sigma^2_{X_B} - \sigma^2_{X_A}} \\
0 &= k^2a^2_{32} + k2a_{22}a_{32} - (\sigma^2_{Y_B} - \sigma^2_{Y_A}) \\
\sigma_{X_B,Y_B} &= \sigma_{X_A,Y_A} + a_{11}ka_{31} + (a_{21}a_{31} + a_{22}a_{32}) + (ka^2_{31} + ka^2_{32} + ka^2_{33}) \\
&= \sigma_{X_A,Y_A} + a_{22}a_{32} + k(\sigma^2_{X_B} - \sigma^2_{X_A})
\end{aligned}$$

Corresponding results can be obtained for the case where: $a_{21}a_{31} + a_{22}a_{32} = 0$.

1.1.3. Negative signal

The above calculations hold for negatively correlated signal. Anti-correlated regions and networks may exist in the brain and many other systems. The anti-correlated signals from these regions are likely to be composed of a combination of negatively and positively correlated signal components (e.g. positively correlated signal noise). Additional noise components will reduce the absolute value of correlations, while more negative correlatino may be produced by increases in the amplitude of the anti-correlated signals. For signals that are negatively correlated overall, a common signal is likely to have opposite effects in both nodes.

1.1.4. Monte-Carlo Inference

To sample the possible distribution of covariance matrices underlying the observations, we assume that their distribution is equal to that of covariance matrices of normally distributed correlated variables sharing the same degrees of freedom (n) (accounting for the autocorrelation in the FMRI data). A $p \times p$ covariance matrix Σ, of Gaussian variables with n degrees of freedom, will have a Wishart distribution, with a pdf:

$$\frac{1}{2^{\frac{np}{2}}|V|^{\frac{n}{2}}\Gamma_p(\frac{n}{2})}|\Sigma_{X,Y}|^{\frac{n-p-1}{2}}e^{-\frac{1}{2}tr(V^{-1}\Sigma_{X,Y})}$$

Where $\Gamma_p()$ is the multivariate gamma function:

$$\Gamma_p(\frac{n}{2}) = \pi^{\frac{p(p-1)}{4}}\pi_{j=1}^{p}\Gamma(\frac{n}{2}+\frac{1-j}{2})$$

Using a flat empirical prior, we used rejection sampling to to obtain samples from the distribution of underlying covariance matrices A and B potentially producing the observed covariances, Q_A, Q_B. For each sample, limits on the range of correlations produced by additive, uncorrelated and common signals can calculated. Additional variability will occur in scenarios with a low initial correlation (or low degrees of freedom), where different samples will have the underlying initial correlation between signals vary in sign.

1.1.5. FMRI Preprocessing

Data were analysed using FSL's FEAT [3], the FSLNets network toolbox [7], and with Python, using SciKits-Learn [4] and the MNE package [1]. Standard FEAT preprocessing was applied, including brain extraction [6], motion correction [2], and no spatial smoothing. To reduce noise, we used our automated denoising tool, FIX (FMRIB's ICA-based Xnoiseifier) [5], which removes artefactual signal components associated with head motion, physics artefacts, and non-neural physiological signals. FIX also integrations a regression of measured head motion parameters. Checks of image quality, head motion, and registration were performed for every scan. Data was high-pass filtered (0.005 Hz) to remove low-frequency drifts and other artefacts that would affect variance estimates. Variance maps and variance change maps were generated using the FSL tools FSLMaths and Randomise. Correlation maps were generated using FEAT and specialised code written in Python.

1.1.6. Modelling of localiser scans

A block-design task activation scan was acquired to identify those regions showing task-related activation to the steady-state tasks investigated here. This involved a nine-minute scanning period that involved randomised 30-second blocks of the visual, motor and motor-visual tasks (total of 12 blocks). The motor-visual attention task was not acquired in this scan. Due to scanner limitations, this scan used a non-accelerated sequence, with a repetition time of 3.0s and 3mm cubic pixel resolution.

A multi-level GLM analyses of the localiser scan was used to identify regions that activated with one or more of the states, relative to rest [3]. The block design time series of each of the three stateswas convolved with a gamma function, and included within a model also included the temporal derivatives of these time series, and motion parameters derived from motion correction. These analyses used temporal autocorrelation correction and outlier detection to ensure model validity (32, 33). Subject-level contrasts were defined for each of the three task states. The resulting parameter maps were registered to standard anatomical space, via their high-resolution structural images using the FSL linear and non-linear registration tools FLIRT and FNIRT. The parameter maps were then combined at the group level in an F-Test to identify brain regions showing positive or negative responses to one or more of the states The maps were transformed into Z-statistic images and thresholded (correcting for multiple comparisons) using FSL FEAT with a cluster-based thresholding approach with cluster-level significance level of $p<0.05$.

1.2. Supplementary results

1.2.1. Supporting model validation and simulations

Supp Fig. 2 shows correlation changes that can be explained by additions of common and uncorrelated signal as a function of initial correlation. Fig. 2A plots scenarios where there are changes in uncorrelated signal in both nodes. If there is no uncertainty in observations, a change unshared signals producing a specific change in variance will produce a specific change in correlation. The resulting correlation scales linearly with the initial level of correlation and with the combined ratios of change in standard deviation of the signals. When observational uncertainty is taken into account, it is possible to identify a band within which correlation would fall if there were a change in unshared signal.

Changes in a common signal component produces the opposite direction of change in correlation (Supp Fig. 2B). Here, a given change variance may cause a range of changes in correlation, depending on the nature of the new signal, S. This range is broader when initial correlation is near zero, as . Note that as the addition of the same signal component to negatively correlated signals these distributions are not symmetric around zero. The range of possible changes is particularly large, including decreases, if

there is a mismatch in the amount of new signal in the processes. Accounting for observational uncertainty broadens the range of observed changes in covariance that can be potentially be explained by a change in a common signal component (Supp Fig. 2).

The addition of distinct signal components (that do not negate existing signal) can produce a wider range of changes in correlation, but the possible changes are still limited by change in variance (Supp Fig. 2C). When initial correlation is near zero, a wide range of changes in correlation can be explained by modest changes in variance. This band widens further when the uncertainty due to limited observations is taken into account Supp Fig.. The potential band of changes in correlation has a nonlinear relationship to the initial correlation, with reductions being greater when the initial correlation is closer to zero. When initial correlation is 0.6, a reduction in variance of 20% in both nodes can explain a complete decorrelation of signals.

1.2.2. *Inference on simulated network changes*

Simulations of signals showing FC changes were generated to match the spectral properties of the empirical dataset described in the present work. Nodes in the initial condition either had a random positive correlation between 0.3 and 0.58 or a correlation of zero, depending on whether nodes were connected. For the second condition, additional signal was added, such that variance of the nodes increased by 20%.

1.3. *Supplementary Figures*

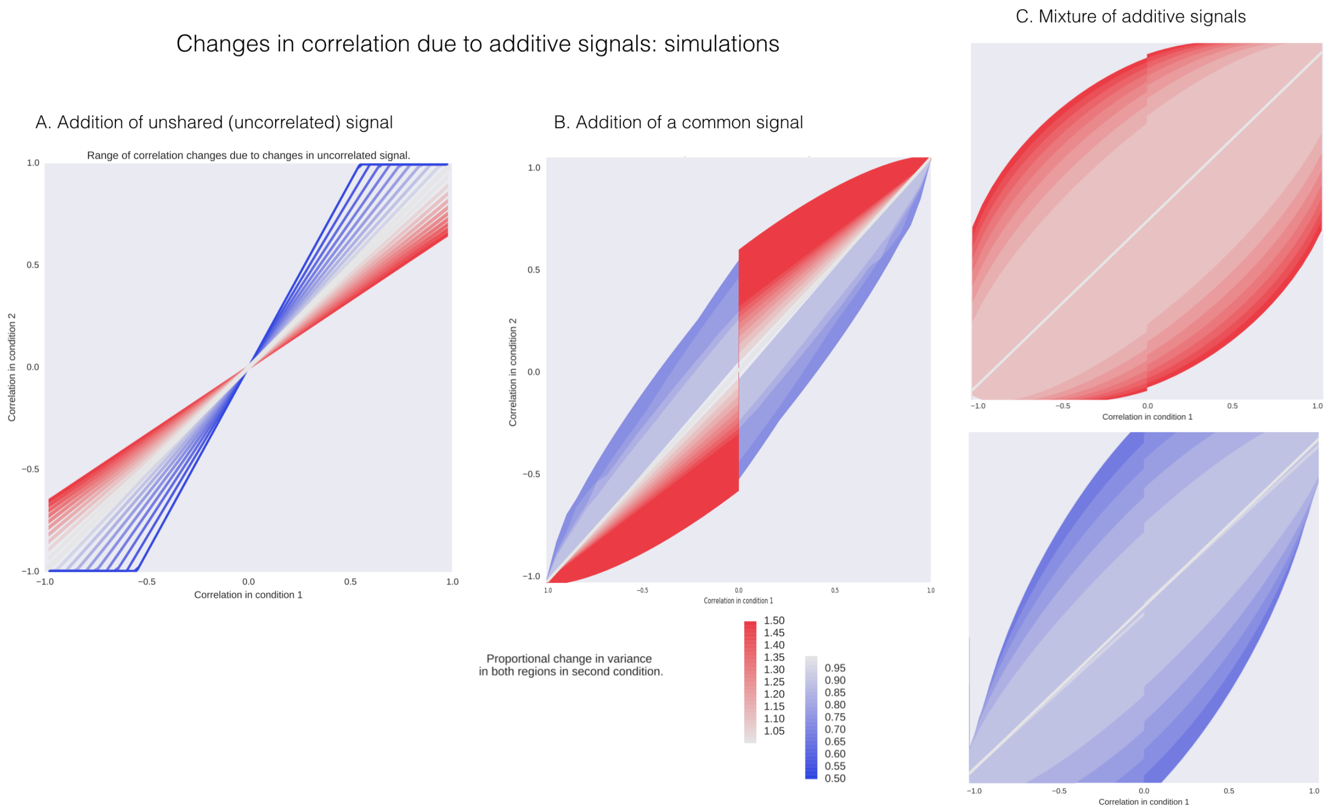

Figure 1: **(Supp.)**The effects on correlation of additive signal producing certain variance changes (not accounting for observation uncertainty). The plots reflect scenarios where both regions receive the same signal. Colour fills indicate the range of levels of correlation in the second condition that could be explained by a specific change in standard deviation. Column **a.** Potential changes associated with additions of uncorrelated signal. Column **b.** Potential changes associated with the addition of a common signal component. Column **c.** Changes associated with a mixture of signal components changing in amplitude.

Table 1: Region label key

Cng	Cingulate Cortex
Cun	Cuneus Cortex
InC	IntraCalcarine Sulcus
SMA	SMA
LOI	Lateral Occipital Cortex (Inferior)
LOS	Lateral Occipital Cortex (Superior)
Lin	Lingual Gyrus
OcP	Occipital Pole
PCu	Precuneus Cortex
TOc	TempOcc
CbL	CbL Cerebellum (Left)
CbR	CbR Cerebellum (Right)
IPL	Inferior Precentral Gyrus (Left)
IPR	Inferior Precentral Gyrus (Right)
PtL	PutL Putamen (Left)
PtR	PutR Putamen (Right)
SMA	SMA
PGL	PostGL Postcentral Gyris (Left)
PGR	PostGR Postcentral Gyris (Right)
PHL	Premotor Cortex Hand area (Left)
PHR	Premotor Cortex Hand area (Right)
LOL	LatOccSL Lateral Occipital Cortex - inferior div. (Left)
LOR	LatOccSR Lateral Occipital Cortex - inferior div. (Right)
LSL	LatOccSL Lateral Occipital Cortex - superior div. (Left)
LSR	LatOccSR Lateral Occipital Cortex - superior div. (Right)
LiL	Lingual Gyrus (Left)
LiR	Lingual Gyrus (Right)
OFR	Occipital: Fusiform Gyrus (Right)
OFL	Occipital: Fusiform Gyrus (Left)
OPL	Occipital Pole (Left)
OPR	Occipital Pole (Right)
TOL	Temporal Occipital (Left)
TOR	Temporal Occipital (Right)

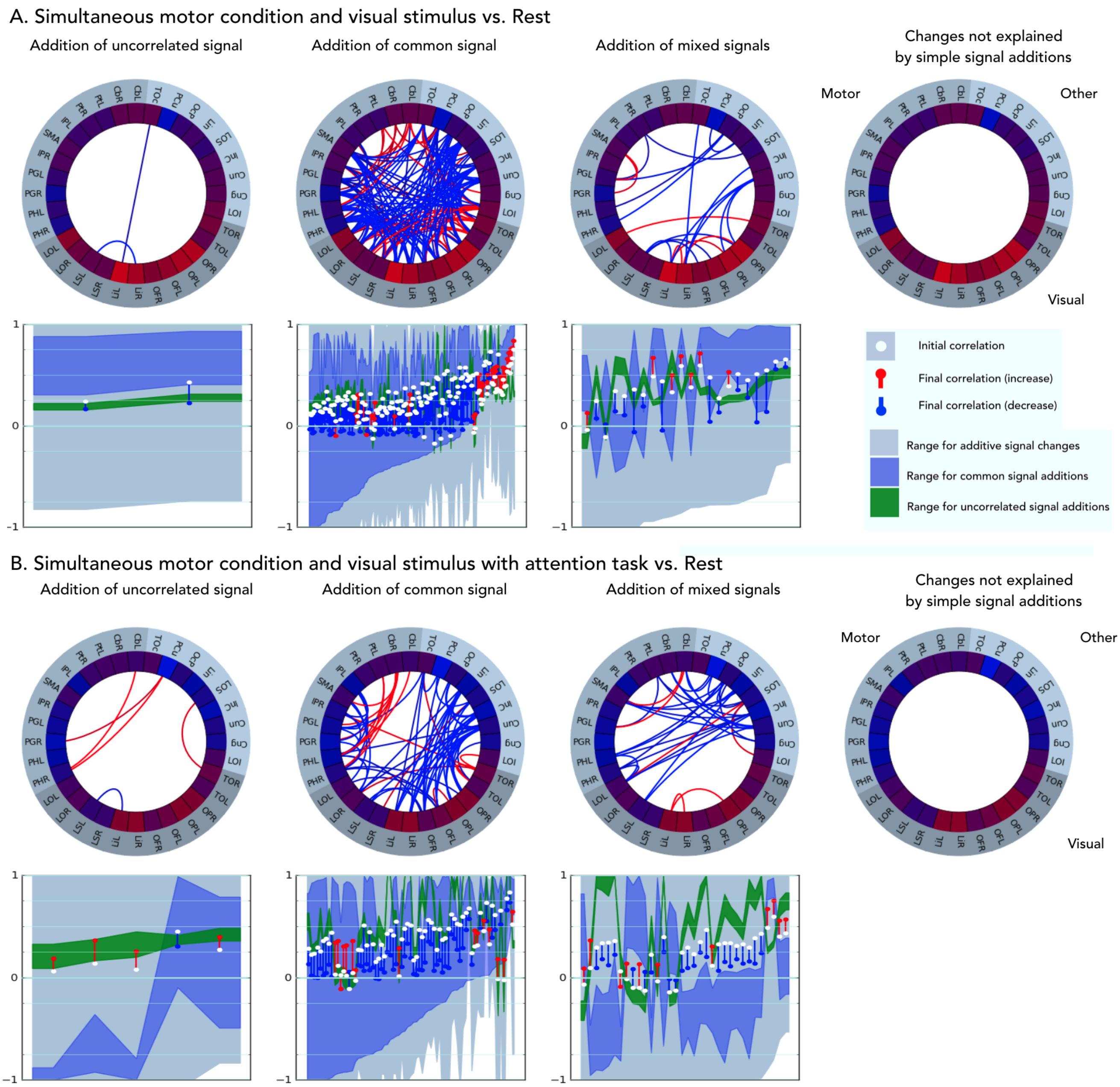

Figure 2: **(Supp.)** Connectivity changes between combined finger tapping and visual stimulus condition and rest, and a more attention demanding combined task and rest. Plot organisation is described in Fig. 5

[1] Gramfort, A., Luessi, M., Larson, E., Engemann, D. A., Strohmeier, D., Brodbeck, C., Parkkonen, L., Hämäläinen, M. S., 2014. MNE software for processing MEG and EEG data. NeuroImage 86, 446–460.

[2] Jenkinson, M., oct 2002. Improved Optimization for the Robust and Accurate Linear Registration and Motion Correction of Brain Images. NeuroImage 17 (2), 825–841.

[3] Jenkinson, M., Beckmann, C. F., Behrens, T. E. J., Woolrich, M. W., Smith, S. M., aug 2012. FSL. NeuroImage 62 (2), 782–90.

[4] Pedregosa, F., Varoquaux, G., Gramfort, A., Michel, V., Thirion, B., Grisel, O., Blondel, M., Prettenhofer, P., Weiss, R., Dubourg, V., Vanderplas, J., Passos, A., Cournapeau, D., Brucher, M., Perrot, M., Duchesnay, E., 2011. Scikit-learn: Machine Learning in {P}ython. Journal of Machine Learning Research 12, 2825–2830.

[5] Salimi-Khorshidi, G., Douaud, G., Beckmann, C. F., Glasser, M. F., Griffanti, L., Smith, S. M., apr 2014. Automatic denoising of functional MRI data: combining independent component analysis and hierarchical fusion of classifiers. NeuroImage 90, 449–68.

[6] Smith, S. M., nov 2002. Fast robust automated brain extraction. Hum. Brain Mapp. 17 (3), 143–155.

[7] Smith, S. M., Miller, K. L., Salimi-Khorshidi, G., Webster, M., Beckmann, C. F., Nichols, T. E., Ramsey, J. D., Woolrich, M. W., jan 2011. Network modelling methods for FMRI. NeuroImage 54 (2), 875–91.
